# Learning alters theta amplitude, theta-gamma coupling and neuronal synchronization in inferotemporal cortex

**DOI:** 10.1186/1471-2202-12-55

**Published:** 2011-06-09

**Authors:** Keith M Kendrick, Yang Zhan, Hanno Fischer, Alister U Nicol, Xuejuan Zhang, Jianfeng Feng

**Affiliations:** 1 Laboratory of Molecular Signalling, Cognitive and Systems Neuroscience Group, The Babraham Institute, Cambridge CB22 3AT, UK; 2 Mathematical Department, Zhejiang Normal University, Zhejiang 321004, PR China; 3 Department of Computer Science, Warwick University, Coventry, CV4 7AL, UK; 4 Centre for Computational Systems Biology, Fudan University, Shanghai, PR China

## Abstract

**Background:**

How oscillatory brain rhythms alone, or in combination, influence cortical information processing to support learning has yet to be fully established. Local field potential and multi-unit neuronal activity recordings were made from 64-electrode arrays in the inferotemporal cortex of conscious sheep during and after visual discrimination learning of face or object pairs. A neural network model has been developed to simulate and aid functional interpretation of learning-evoked changes.

**Results:**

Following learning the amplitude of theta (4-8 Hz), but not gamma (30-70 Hz) oscillations was increased, as was the ratio of theta to gamma. Over 75% of electrodes showed significant coupling between theta phase and gamma amplitude (theta-nested gamma). The strength of this coupling was also increased following learning and this was not simply a consequence of increased theta amplitude. Actual discrimination performance was significantly correlated with theta and theta-gamma coupling changes. Neuronal activity was phase-locked with theta but learning had no effect on firing rates or the magnitude or latencies of visual evoked potentials during stimuli. The neural network model developed showed that a combination of fast and slow inhibitory interneurons could generate theta-nested gamma. By increasing N-methyl-D-aspartate receptor sensitivity in the model similar changes were produced as in inferotemporal cortex after learning. The model showed that these changes could potentiate the firing of downstream neurons by a temporal desynchronization of excitatory neuron output without increasing the firing frequencies of the latter. This desynchronization effect was confirmed in IT neuronal activity following learning and its magnitude was correlated with discrimination performance.

**Conclusions:**

Face discrimination learning produces significant increases in both theta amplitude and the strength of theta-gamma coupling in the inferotemporal cortex which are correlated with behavioral performance. A network model which can reproduce these changes suggests that a key function of such learning-evoked alterations in theta and theta-nested gamma activity may be increased temporal desynchronization in neuronal firing leading to optimal timing of inputs to downstream neural networks potentiating their responses. In this way learning can produce potentiation in neural networks simply through altering the temporal pattern of their inputs.

## Background

The functions of both low and high frequency oscillations in the brain are the subject of considerable speculation [1]. Low frequency theta oscillations (4-8 Hz) have been observed to increase in terms of power during working memory tasks [2,3] and in power and phase-locked discharge of single neurons in a visual memory task [4]. In hippocampus the phase of theta functions as the clock signal for timing of pyramidal neurons and long-term potentiation (theta peaks) and depotentiation (theta troughs) [5]. These findings may reflect the patterns of synaptic plasticity and maintenance of the memory for a stimulus. Fast frequency gamma oscillations (30-70 Hz) can provide tighter control and coordination than lower frequency ones [6] and are hypothesised to be responsible for higher cognitive functions such as perceptual binding of visual features [7]. Human electroencephalographic (EEG) recordings show event-related gamma activity indicating gamma as a signature of cortical networks underlying object representations[8]. Modulation of oscillatory synchronization can also increase synaptic gain at postsynaptic target sites thereby potentiating responses to learned stimuli [9,10].

Coupling between gamma amplitude and theta phase (theta-nested gamma) has been reported in both cortex and hippocampus [1,11-13] and provides an effective combination for neuronal populations to communicate and integrate information during visual processing and learning. It may also provide a process of temporal segmentation that can maintain multiple working memory items [14]. Altered coupling has been reported both in the context of human cognitive and perceptual tasks in the cortex [11] and in the rat hippocampus during item-context association learning [12], although how this might act to modulate neuronal activity has yet to be established.

There is still debate as to whether functionally important changes in theta or gamma involve amplitude or phase parameters, or both. Some studies report that theta phase rather than amplitude is correlated with cognitive processes, the so-called phase reset model [1,15,16], while others place more importance on coupling between theta amplitude and gamma frequency [11,12]. The magnitude of both theta and gamma oscillations during encoding also appears to predict the efficacy of subsequent recall [17] and theta can both modulate gamma amplitude [18] and the firing of single neurons [4]. The ratio of theta to gamma power has also recently been shown to be correlated with memory function in humans [19]. It is clearly important therefore that changes in different theta and gamma parameters are investigated in a number of different learning contexts to help establish some general principles and also to aid development of neural network models which can further inform our interpretation of the outcome of these changes on neural encoding.

Face recognition learning is known to involve the inferotemporal cortex in humans, monkeys and sheep [20] and there is phase locking between neuronal activity and theta in this region in humans [21]. We have therefore investigated the effects of face and object discrimination learning on theta and gamma oscillations and coupling and neuronal activity in sheep IT using 64-electrode recording arrays. Our results have identified learning-related changes in the amplitude of theta, the theta/gamma ratio and the coupling between theta phase and gamma amplitude. We have therefore also developed a neural network model which can effectively reproduce our electrophysiological findings. This model predicted that a consequence of these learning evoked changes in theta amplitude and theta-gamma coupling would be a potentiation of the firing of downstream neurons by desynchronizing the firing of excitatory neurons projecting to them. The presence of this predicted desynchronization effect following learning was then confirmed in multiunit activity recordings from the IT.

## Results

### Visual discrimination performance during recordings

Overall local field potential and MUA data were collected from 51 separate blocks (Sheep A: 17, B: 24, C: 10) of visual discrimination trials (20-60 trials per block). During these trials the sheep were each presented with a total of 4 to 10 different face pairs and in addition two sheep were each presented with a non-face object pair (see Additional file 1, Figure S1). Successful learning was defined as the first block of 20 trials during which the animal achieved > 80% and then continued subsequently to perform at or above this criterion. To compare different electrophysiological parameters as a function of learning blocks of trials for each animal were sub-divided on the basis of whether the > 80% correct learning criterion for a particular face or object pair had been achieved or not. Respective mean ± sem discrimination performances on trial blocks during and after learning were: Sheep A: 57.8 ± 3.5% vs 89.4 ± 3.1%; Sheep B: 58.7 ± 8.5% vs 89.4 ± 1.5%; Sheep C: 66.8 ± 8.5% vs 90.5 ± 2.5%. There were no significant differences in response times made by the three animals during trials with errors as opposed to correct choices. After learning, while there was a slight tendency for response times to be shortened this was not significant in any animal. Respective mean ± sem response times for both correct and error choices on trial blocks during and after learning were: Sheep A: 1.89 ± 0.06s (correct) and 1.87 ± 0.09s (error) vs 1.77 ± 0.06s (correct) and 1.73 ± 0.04 (error); Sheep B: 2.42 ± 0.10 (correct) and 2.35 ± 0.10s (error) vs 2.31 ± 0.09s (correct) and 2.48 ± 0.12s (error); Sheep C: 2.99 ± 0.38s (correct) and 3.44 ± 0.38s (error) vs 2.37 ± 0.11s (correct) and 2.38 ± 0.26s (error) - p > 0.05 in all cases). This also indicates that in general the animals were equally motivated to perform the task during and after learning.

Following training, sheep generally learned to discriminate between new pairs of faces at > 80% correct in 20-80 trials although this was highly variable and learning could occur over time-periods of anything from 5-10 minutes to several days or more. In three cases (two in Sheep A and one in Sheep B) the > 80% criterion for novel face pairs was not reached even after 80-140 trials conducted across several days of recording sessions.

### Theta and gamma oscillations in inferotemporal cortex

**Figure 1:**
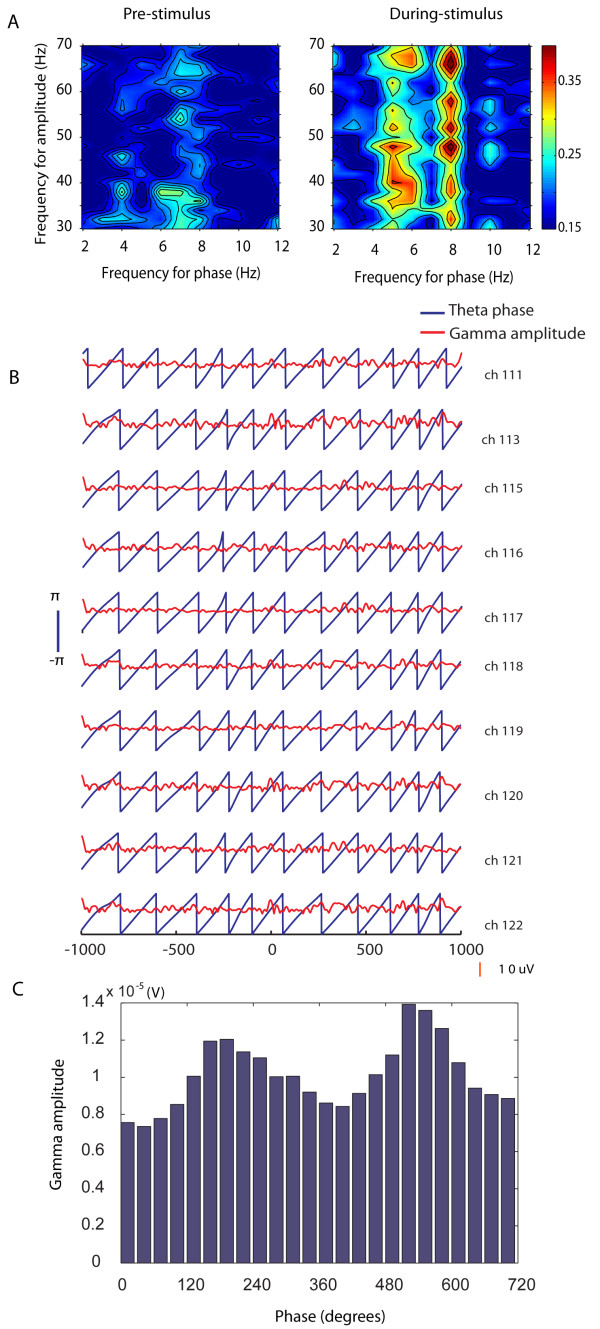
**Theta-gamma coupling in IT**. (A) shows a typical example of coupling between theta phase and gamma amplitude taken from Sheep B (session 110305-1, channel 3) for the pre-stimulus (left) and during-stimulus (right) periods before (left) and during (right) presentation of a learned stimulus pair. There is a clear increase in coherence (represented by pseudocolor scale) across the entire gamma range and parts of the theta range (particularly at 8 Hz) and no evidence for coupling with gamma at phase frequencies immediately below or above the theta band. The red/yellow areas indicate where coupling between the two frequencies was significant (p < 0.001). (B) Shows typical data from 10 adjacent individual channels (from same sheep and recording session as in (A)) which exhibited significant (p < 0.001) coupling between theta phase and gamma amplitude across a 1 s before and a 1s period during stimulus presentation. Peaks in gamma amplitude (red) can clearly be seen across the electrodes during the majority of the theta cycles displayed. These data also illustrate the strong synchronization of theta phase across individual channels. Patterns of gamma amplitude changes are also very similar across the electrodes. (C) Shows an example of the strength of coupling between theta phase and gamma amplitude for a single channel across two theta cycles. It can be seen that there is consistently stronger locking at around 180 degrees in both cycles.

A wavelet transform applied to each individual LFP showed substantial theta band activity across the 4-8 Hz range, and synchronized across IT electrodes, before and during stimulus presentation (see Figure 1 and Additional file 1, Figure S2). There was a much smaller contribution from gamma band activity (30-70 Hz) and across the recording sessions significant (p < 0.001) coupling occurred between theta phase and gamma amplitude both before (mean ± sem = 76.5 ± 6.2% of recording electrodes in left IT and 84.6 ± 4.2% in right IT) and during (80.6 ± 5.8% in left IT and 84.6 ± 4.7% in right IT) visual stimulus presentation (see Figure 1). While we were able to detect significant power in the high gamma range (70-100 Hz) in some recording sessions it was extremely low (15-25% of low-gamma power) and we therefore focussed our analyses on the low gamma range. Where high gamma power was significantly above noise we found that its amplitude showed a similar degree of coupling with theta phase across the 70-100 Hz range as for low frequency gamma (data not shown).

### Effects of learning on theta and gamma oscillations

**Figure 2:**
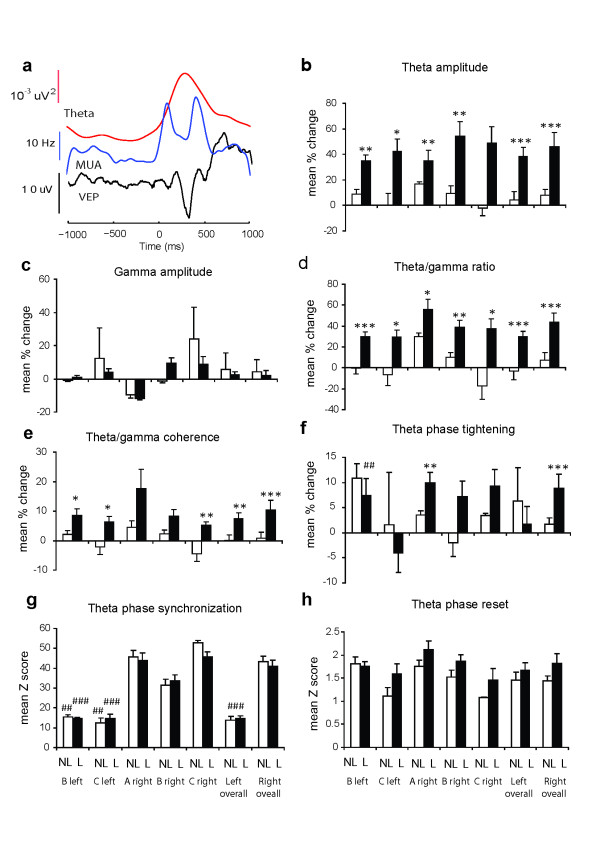
**Evoked potential, MUA and theta latency profiles and theta/gamma activity changes after learning**. (A) Typical average latency profile for theta, MUA and VEP (P100 and N300) over 40 trials post learning (face-pair shown at time 0). The change in MUA in response to the stimulus is mainly restricted to the 500 ms time window. (B) Mean ± sem % change in theta amplitude (C) Gamma amplitude (D) Theta/gamma ratio (E) Theta gamma coherence and (F) Theta phase tightening (G) Mean ± sem z-scores for theta phase synchronization and (H) Theta phase reset overall and in each of the 3 different animals (A, B and C), and for both the right and left IT, during sessions where discrimination learning performance had yet to reach > 80% (NL = not learned) compared with those where it had (L = learned - corrected t-tests, *p < 0.025 - left IT, and < 0.017 for right IT, **p < 0.01, ***p < 0.001 vs NL and ##p < 0.01, ###p < 0.001 for left IT vs right IT). For overall changes across the sheep p < 0.05, **p < 0.01 and ***p < 0.001 from ANOVA tests. For both NL and L blocks, comparisons were made between data averaged across electrodes and individual trials for the 500 ms immediately prior to stimulus presentation and the first 500 ms during it.

In all cases effects of learning were tested using ANOVA tests but in a number of cases data for did not show a normal distribution and so p values from an additional permutation test (PT) are also given where these were significant or close to significance. An analysis of theta wave activity across the three animals revealed a significant increase following learning in theta amplitude (two-way ANOVA: left IT, *F_1,29 _*= 20.0, p < 0.001; right IT, *F_1,41 _*= 18.2, p < 0.001; PT, p < 0.001 in both cases) during the first 500 ms after stimulus onset compared with the 500 ms prior to it. A 500 ms time window was chosen throughout for the analysis of learning effects since animals were capable of making an operant response in 1s in some cases and could only be guaranteed to be looking at the stimulus pictures for ~500 ms. We wanted to limit our analyses to the perceptual processing component of the task rather than to the response phase. The observed changes in theta amplitude during this period represented an increase of 20-50% in each animal following learning (Figure 2B). The proportion of recording electrodes showing a significant (p < 0.05) during-stimulus rise in theta amplitude increased in all animals after learning and in both left and right IT (left IT: *F_1,29 _*= 13.87, p < 0.001 - mean ± sem during learning = 2.51 ± 1.49% vs 42.26 ± 8.92% of electrodes after learning; right IT: *F_1,41 _*= 39.03, p < 0.001 - during learning = 3.19 ± 2.35% vs 64.47 ± 12.43% of electrodes after learning; PT, p < 0.001 in all cases). During learning the proportion of electrodes showing increased theta amplitude during stimulus presentation did not differ significantly from chance (one-sample t-test, left IT t^11 ^= 1.96, p = 0.076; right IT t^21 ^= 1.45, p = 0.163). There were no overall significant changes in gamma amplitude (left IT: *F_1,29 _*= 0.719, p = 0.404; right IT: *F_1,41 _*= 0.375, p = 0.544 - see Figure 2C) as a result of learning, and no individual electrodes showed a significant increase in gamma amplitude. The net result was a significant 25-55% increase in the ratio of theta to gamma amplitude (left IT: *F_1,29 _*= 24.08, p < 0.001; right IT: *F_1,41 _*= 31.22, p < 0.001; PT, p < 0.001 in all cases) during stimulus presentation after learning (see Figure 2D).

In addition, coupling between theta and gamma was also strengthened after learning in terms of a greater coherence (~7%) between the two frequencies (left IT: *F_1,29 _*= 9.35, p = 0.005, PT, p < 0.001; right IT: *F_1,41 _*= 14.4, p < 0.001, PT, p = 0.0011)(see Figure 2E). A significant effect of learning was also seen using the mean vector length method [11] (left IT: mean ± sem change during learning = 1.2 ± 10.1%, after learning = 11.1 ± 3.01% - *F_1,29 _*= 4.37, p = 0.04, PT, p = 0.0189; right IT: mean ± sem change during learning = - 0.93 ± 5.3%, after learning = 22.1 ± 6.64% - *F_1,41 _*= 11.19, p = 0.002, PT, p < 0.001 ) and modulation index method [13] (left IT: mean ± sem change during learning = 0.6 ± 7.07%, after learning = 26.0 ± 8.04% - *F_1,29 _*= 4.39, p = 0.04, PT, p = 0.003; right IT: mean ± sem change during learning = 0.9 ± 4.67%, after learning = 28.8 ± 8.15% *F_1,41 _*= 9.83, p = 0.003, PT, p < 0.001) for quantifying coupling strength. There was also significantly increased tightening of theta phase across electrodes after learning in the right IT (left IT: *F_1,29 _*= 0.91, p = 0.348; right IT: *F_1,41 _*= 8.09, p = 0.007, PT, p = 0.0014 - see Additional file 1, Figure S8a) where z-scores were ~3-fold higher in the right IT than in the left IT (see Figure 2F). A 3-way ANOVA adding hemisphere as a factor showed that while there was no overall effect of side (*F_1,56 _*= 0.03, p = 0.871) or learning (*F_1,56 _*= 0.24, p = 0.629) there was a small significant interaction between learning and hemisphere (*F_1,56 _*= 3.89, p = 0.05) indicating that learning was affecting phase tightening differentially in the left and right IT.

We found no evidence for extensive theta-phase resetting in response to stimulus presentation with < 1.5% of recording electrodes showing a significant (p < 0.05) effect. There was also no significant increase in the associated phase reset z-score following learning in the left IT (*F_1,29 _*= 1.30, p = 0.263), although in the right IT significance was just achieved with the ANOVA but not the permutation analysis (*F_1,41 _*= 5.00, p = 0.031, PT, p = 0.062). However, these mean z-scores for theta phase reset were generally very low (from 1.5-2.1) (Figure 2G).

Overall, levels of theta synchronization across recording electrodes were higher in the right IT (> 95%) than in the left (~48%) (3-way ANOVA: *F_1,56 _*= 189.7, p < 0.001, PT, p < 0.001) but with no effect of learning in either hemisphere (left IT: *F_1,29 _*= 0.34, p = 0.657; right IT: *F_1,41 _*= 0.55, p = 0.561) (see Figure 2H).

### Correlations between altered theta and gamma oscillations and behavior

With only a relatively small number of blocks of trials being recorded in each animal for the main analysis both overall correlations across the three sheep and individual correlations were performed. Table 1 shows that In both left and right IT there was a significant positive correlation between behavioral discrimination performance in each block of trials and the theta and gamma parameters influenced by learning (theta amplitude; theta-gamma ratio; theta-gamma coherence) both across the 3 sheep and in the majority of cases in each individual A similar correlation with behavioral performance was also found when strength of theta-gamma coupling was quantified using the mean vector length method [11] or modulation index method [13]. There was a trend toward a positive correlation with theta phase tightening in the right IT but this just failed to achieve significance either overall (p = 0.06) or in any individual animal.

**Table 1:** Correlations between theta and gamma parameters in IT and discrimination performance

Theta and gamma	Sheep A	Sheep B	sheep B	Sheep C	Sheep C	Overall	Overall
parameters	Right IT	Left IT	Right IT	Left IT	Right IT	Left IT	Right IT
	n = 17	n = 24	n = 22	n = 10	n = 10	n = 34	n = 49

Theta amplitude	+0.56*	+0.51*	+0.41*	+0.62*	+0.59	+0.54**	+0.45**
Gamma amplitude	-0.26	+0.25	+0.33	-0.28	-0.43	-0.04	+0.19
Theta/gamma ratio	+0.59*	+0.76*	+0.37	+0.72*	+0.72*	+0.75**	+0.38**
Theta phase tightening	+0.45	+0.00	+0.25	-0.32	+0.30	-0.11	+0.26

Theta/gamma coupling parameters							
Coherence	+0.50*	+0.40*	+0.41*	+0.53	+0.75*	+0.39**	+0.37**
Mean vector length	+0.45	+0.59*	+0.53*	+0.63*	+0.58	+0.56**	+0.45**
Modulation index	+0.47*	+0.58*	+0.38	+0.50	+0.42	+0.51**	+0.35*

*p =/< 0.05, ** p < 0.01 - Pearson correlation test

**Figure 3:**
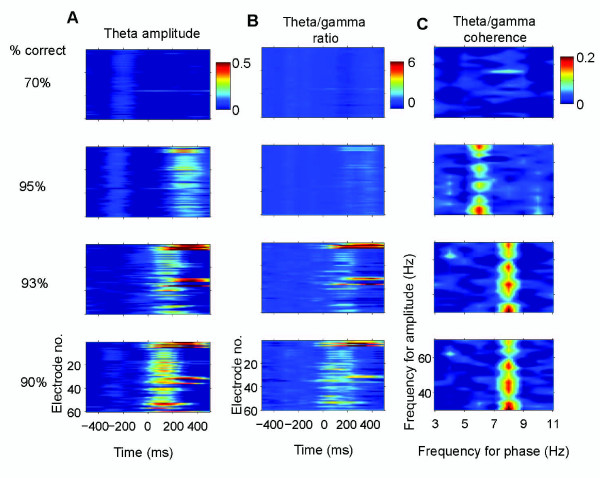
**Rapid time course of learning effects on theta nested gamma**. Pseudocolor panels show changes in: (A) Theta amplitude, (B) Theta/gamma ratio and (C) Coherence between theta phase and gamma amplitude in the right IT during the learning of one new face pair in Sheep B over sequential (top to bottom) blocks of 20 trials conducted over approximately a 20 min period (data plotted from 60 electrodes). Discrimination performance in each of the 4 blocks is shown on the left hand side (i.e. the learning criterion of > 80% correct was achieved in the second block of 20 trials and in subsequent blocks). The face pair stimulus occurs at time zero and the pseudocolor scale indicates normalised (by the maximum value during the stimulus) differences between pre and during stimulus. It can be seen that increased theta amplitude and the theta/gamma ratio occur in blocks of trials where the learning criterion is achieved. Theta-gamma coupling also increases across the gamma range but most notably at 6 and 8 Hz in the theta range.

**Figure 4:**
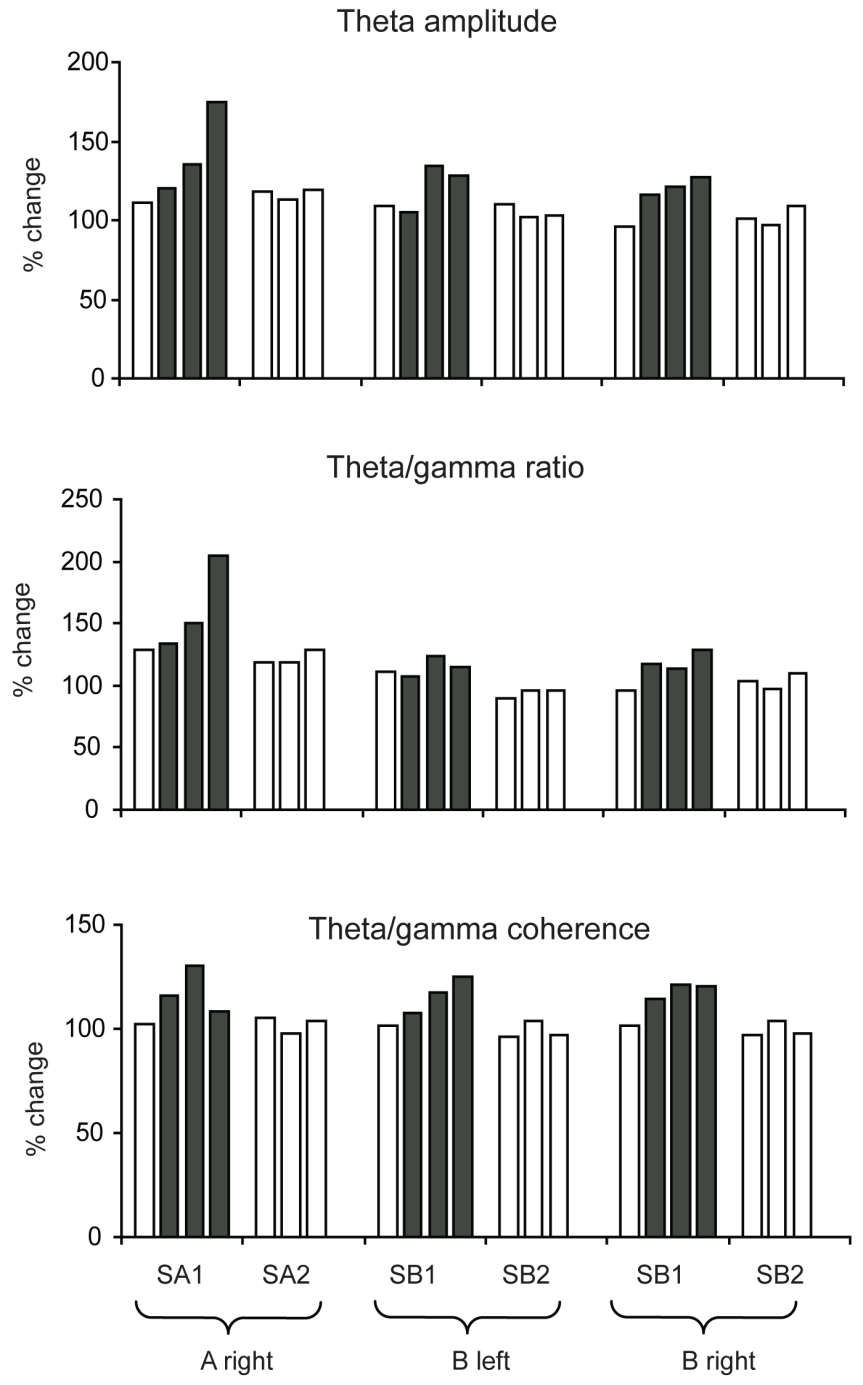
**Theta/gamma changes only occur across sequential blocks of trials where learning occurs**. Histograms show examples of % change in theta amplitude, the theta/gamma ratio and theta/gamma coherence across sequential blocks of trials (either 3 or 4 blocks of 20-40 trials) for novel face-pair discriminations where two sheep either do (SA1 and SB1) or don't (SA2 and SB2) achieve a > 80% learning criterion. Blocks of trials where the > 80% criterion was achieved are shown as black bars. It can be seen that there is generally a very good correspondence between increases in these three parameters and performance above the learning criterion being achieved and maintained. There is no suggestion of changes occurring simply as a function of repeating blocks of trials over a particular time course and independent of discrimination performance

In the majority of cases we were unable to make recordings where an animal learned to discriminate between a specific face-pair over successive blocks of trials during the same recording session, although we were able to do this on one occasion for both sheep A and B. Figure 3 shows data for Sheep B which clearly illustrate that increased theta amplitude, theta/gamma ratio and theta/gamma coherence occurred immediately in the first block of 20 trials where the > 80% correct criterion was reached. Thus changes in these parameters could take place in 5-10 min of trials although in most cases they were observed over trials conducted across several days. We also confirmed that in four other cases (three for sheep A and one for Sheep B), where 80-140 successive trials were given with the same face pair but the learning criterion was not achieved, there was no corresponding change in these same theta and gamma parameters. This showed that the observed learning effects were not simply due to stimulus repetition (see Figure 4).

Finally, for the two learned face-pairs where we ran additional blocks of trials in Sheep A and B with the face images inverted, this resulted in a complete inability to discriminate between the two faces (55% and 45% correct responses respectively). As expected, the patterns of theta/gamma changes in both cases were similar to those seen in the face pairs prior to the learning criterion being achieved (data not shown).

### Interdependence of theta amplitude and theta-gamma coupling changes

**Figure 5:**
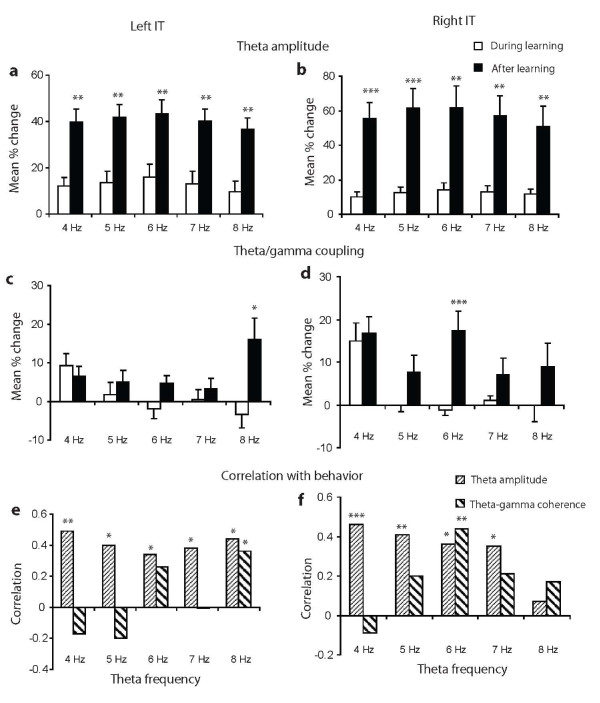
**Differential effects of learning on theta amplitude and theta-gamma coupling**. (A-D) histograms show effects of learning on theta amplitude and theta-gamma coupling where the theta band is divided up into 1 Hz steps from 4-8Hz: (A) Theta amplitude in left IT, (B) Theta amplitude in right IT, (C) Theta-gamma coupling in left IT, (D) Theta-gamma coupling in right IT, In (E-F) histograms show correlations with discrimination performance: (E) Correlation with theta amplitude and theta-gamma coherence in the left IT, (F) same as (E) but for the right IT. *** p < 0.001, ** p < 0.01 and * p < 0.05 vs during learning (A-D) or correlation with behavior (E-F).

Since it was possible that theta-gamma coupling changes might be totally dependent upon those of theta amplitude, and both had a similar positive correlation with discrimination performance, we carried out a number of further analyses to test whether theta amplitude changes were always predictive of those in theta-gamma coupling. Firstly, we found no evidence for a significant positive correlation between theta amplitude and the strength of theta-gamma coherence across the 3 animals (left IT: r = 0.30 and right IT: r = 0.14, p > 0.05 in both cases). Next, we carried out an additional analysis looking at changes in these two parameters across different individual theta frequencies. While learning significantly increased theta amplitude at all 1 Hz intervals across the 4-8 Hz theta range in both left and right IT (Figures 5A and 5B), theta-gamma coherence was only significantly increased at 8 Hz in the left IT and 6 Hz in the right IT (Figures 5C and 5D). There were also no significant positive correlations between them at any individual theta frequency (left IT: 4 Hz r = -0.30, 5 Hz r = 0.03, 6 Hz r = 0.10, 7 Hz r = -0.09, 8 Hz r = 0.25; right IT: 4 Hz r = 0.07, 5 Hz r = 0.12, 6 Hz r = 0.13, 7 Hz r = 0.04, 8 Hz r = -0.03, p > 0.05 in all cases). This restricted involvement of higher theta frequencies in increased coupling with gamma following learning can also be seen in Figure 3. Interestingly, equivalent significant stimulus-evoked increases in theta-gamma coherence occurred at 4 Hz both during and after learning (Left IT: during learning, t^11 ^= 3.35, p = 0.006, after learning, t^21 ^= 2.98, p = 0.007; Right IT: during learning, t^21 ^= 3.04, p = 0.006, after learning, t^25 ^= 3.88, p < 0.001), Thus, visual stimuli routinely increase coupling between the phase of low frequency theta and gamma amplitude but learning is specifically associated with increased coupling of gamma amplitude and theta phase at higher theta frequencies. Analysis of correlations between behavior and theta amplitude within the three animals showed a consistent pattern between them (i.e. positive correlations were generally high across the entire theta frequency range). However, with theta-gamma coupling Sheep A in the right IT had the strongest positive correlations with behavior only at 6 (0.67) and 7(0.58)Hz. Sheep B had strongest positive correlations in both hemispheres at 6 (right 0.33, left 0.35) and 8 (right 0.40, left 0.32)Hz while Sheep C a strong correlation in both hemispheres only at 8 Hz (right 0.50, left 0.64).

### Visual evoked potentials and MUA responses

Following stimulus onset there were similar peak response latencies for the VEP, MUA and peak theta amplitude (overall mean ± sem across all recording sessions in the 3 animals: VEP: P100 = Right 133 ± 7 ms, Left = 118 ± 6 ms; N300 = Right 367 ± 13 ms Left 321 ± 15 ms; MUA: Right 266 ± 4 ms, Left 255 ± 4 ms; Theta = Right 265 ± 14 ms, Left 234 ± 11 ms) (see Figure 2A). However, as would be predicted from the lack of evidence for theta phase resetting in response to stimulus presentation, there was no significant correlation between the latency of peak theta amplitude and that of the MUA (left IT: r = 0.164; right IT: r = -0.02, P > 0.05 in both cases) or the N300 (left IT: r = 0.35; right IT = 0.16, P > 0.05 in both cases) although there was with the P100 in the left (r = 0.49, p = 0.006) but not the right IT (r = 0.27, p = 0.08). The patterns of the VEPs were visibly different between face and object pairs where the magnitude of the P100 component was consistently greater for face pairs than for non-face objects and face inversion also reduced its size compared to when upright faces were presented (see Additional file 1, Figure S4). Neither the response latencies nor the magnitudes of the P100 and N300 components of VEPs were influenced by learning (response latencies: P100 - excluding the two non-face pairs: left IT: during learning = 111.1 ± 7.4 ms vs after learning 123.4 ± 8.2 ms, *F_1,27 _*= 0.383, p = 0.541; right IT: during learning = 131.8 ± 5.9 ms vs after learning 132.7 ± 11.9 ms, *F_1,27 _*= 0.054, p = 0.817, N300: left IT: during learning = 310.2 ± 24.3 ms vs after learning 327.6 ± 19.9 ms, *F_1,29 _*= 0.012, p = 0.914; right IT: during learning = 380.3 ± 16.6 ms vs after learning 355.8 ± 19.9 ms, *F_1,41 _*= 0.178, p = 0.675, response magnitude: P100: left IT: during learning = 5.56 ± 0.09 μV vs after learning = 7.29 ± 1.5 μV, *F_1,27 _*= 0.559, p = 0.461; right IT: during learning = 5.0 ± 0.9 μV vs after learning = 7.3 ± 1.4 μV, *F_1,37 _*= 0.241, p = 0.627, N300: left IT: during learning = -12.52 ± 3.0 μV vs after learning = -15.04 ± 2.37 μV, *F_1,29 _*= 0.328, p = 0.541; right IT: during learning = -11.5 ± 1.4 μV vs after learning = -14.01 ± 1.83 μV, *F_1,41 _*= 0.67, p = 0.418).

A number of MUA recording channels showed significant (p < 0.05) phase locking with theta in each block of visual discrimination trials (during learning mean ± sem % across the theta range = left IT: 20.4 ± 2.4%; right IT 19.7 ± 1.7% and after learning = left IT: 30.7 ± 2.4%; right IT: 25 ± 2.4%). The slight increase after learning was significant in the left (*F_1,26 _*= 4.91, p = 0.036, PT, p = 0.022) but not in the right IT (*F_1,38 _*= 1.95, p = 0.171). Phase locking occurred in each 1 Hz bandwidth from 4-8 Hz frequencies both during and after learning (see Additional file 1, Figure S5).

**Figure 6:**
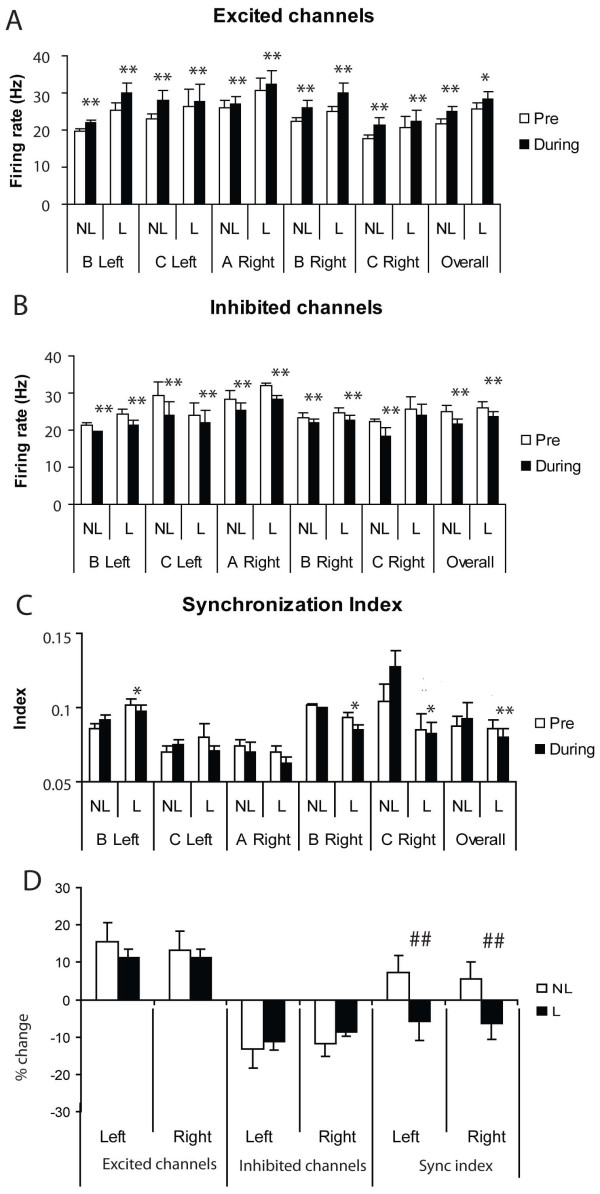
**Effects of learning on MUA firing rates and synchronization**. (A) mean ± sem change in firing rate in response to visual discrimination stimuli in recording channels showing an overall increase in firing rate during the stimulus across a blocks of trials where either the animals (Sheep A-C and either left or right IT) were still learning (NL = not learned) or had learned (> 80% correct choice, L = learned)(open bars are pre-stimulus and black bars during stimulus values). The overall mean for all animals is shown on the right hand side of each set of histograms. (B) Similar changes in firing rates of recording channels showing reduced firing rates. (C) Change in synchronization index of neuronal firing as a function of learning. Paired t-test: * p < 0.05, ** p < 0.01 for the 500 ms pre vs 500 ms during the stimulus. (D) Shows overall % changes across the 3 animals as a function of learning with significant effects of learning revealed by ANOVA tests only for the synchronization index in both left and right IT (## p < 0.01).

For analysis of MUA firing rate changes electrode channels were subdivided into those that either increased or decreased their mean firing frequency during a block of trials. As can be seen from the example MUA response profile given in Figure 2B in most cases the duration of altered firing rates in response to stimuli did not extend beyond the 500 ms sampling period chosen for analysis. Overall, there was no significant effect of learning on the number of recording channels showing increased or decreased firing rates (Mean ± sem % channels excited in Left IT: 60.3 ± 6.8% during learning and 55.7 ± 4.4% after learning, *F_1,27 _*= 0.442, p = 0.512; Right IT: 43.2 ± 11.7% during learning and 48.6 ± 13.1% after learning, *F_1,39 _*= 0.78, p = 0.382) and no effect on the magnitude of the changes in firing rate shown in response to visual stimuli (see Figure 6). This absence of learning-associated firing rate changes is consistent with the similar absence of changes in gamma amplitude.

### Theta-nested gamma generated by a neuronal network

**Figure 7:**
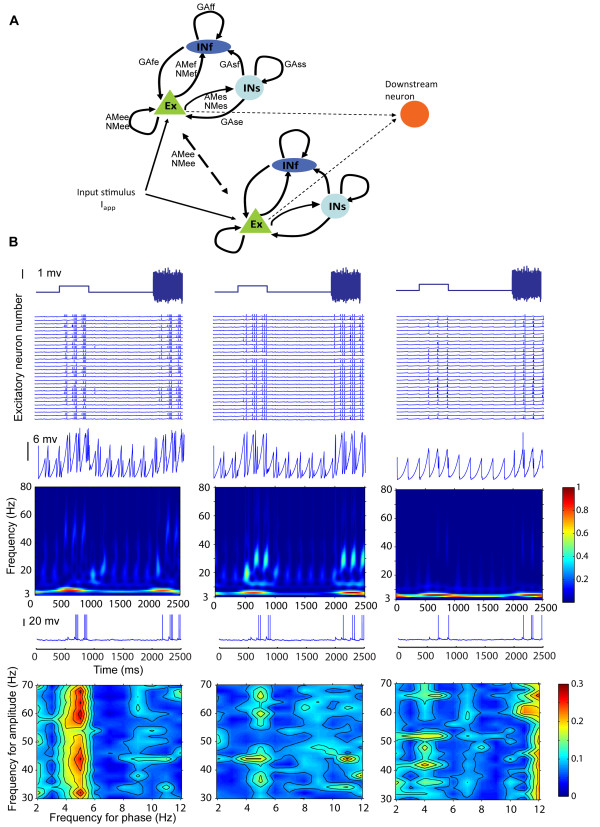
**Neural network model and simulations showing effects of altering theta and gamma contributions on excitatory output neuron and downstream neuron activity**. (A) Schematic showing connectivity in the neural network model together with the coefficient variables representing AMPA (AM), NMDA (NM) and GABA_A _(GA) receptors (INs = slow and INf = fast inhibitory neurons, Ex = excitatory neurons; lower case letters indicate direction of connectivity - s = INs, f = INf and e = Ex - i.e. NMes = NMDA receptor activated by connection from Ex to INs neurons). (B) Responses to both a ramped and white noise stimulus (top. I_app _= 0.8) (top panel) made by excitatory neurons (second panel)), LFP (third panel), the power contribution of different frequencies across the theta/gamma range (fourth panel), the downstream neuron (fifth panel) and coupling of theta phase and gamma amplitude (bottom panel). Data in the three columns are from a single run of the model using different parameter settings (Left, shallow theta-nested gamma with a theta/gamma ratio 3.4:1; middle, deep theta-nested gamma with a theta/gamma ratio 2.7:1; right, minimal gamma with a theta/gamma ratio of 10:1).

We next generated a neural network model of a similar size to the typical number of single neurons sampled by our 64-channel arrays (~200). The model has 100 excitatory (glutamatergic with α-amino-3-hydroxyl-5-methyl-4-isoxazole-propionate (AMPA) and N-methyl-D-aspartic acid (NMDA) receptors) output neurons modulated by 50 fast and 50 slow inhibitory (γ-aminobutyric acid type A receptors (GABA_A_) neurons and projecting to a single downstream neuron (Figure 7A). By adjusting the coupling strength between these neurons we found they could indeed produce theta-nested gamma oscillations (Figure 7B). The generation of theta nested gamma required only a weak, but present, coupling coefficient between the fast inhibitory GABA_A _receptor type neurons and the excitatory neurons and a strong coupling between the latter and the slow inhibitory type ones. There also had to be recurrent coupling between the fast inhibitory and excitatory cells. Increasing the fast inhibitory coupling strength tended to amplify gamma activity whereas increasing that of the slow inhibitory coupling amplified theta. So the two types of connections appear competitive in this context.

### Effects of altering NMDA receptor sensitivity in the model

We first established that the model was able to reproduce patterns of theta and gamma activities observed in the IT. Simulations revealed similar changes in theta power during stimulus application and at the same latency (see Additional file 1, Figure S6 and Additional file 1, Figure S7). Theta activity was also strongly synchronised across the network and there was phase tightening during stimulus presentation (see Additional file 1, Figure S7 and Additional file 1, Figure S8).

Having validated the model's utility we next used it to investigate potential functional consequences of altering the ratio of theta to gamma to produce shallow nested gamma (as seen after learning) on communication between excitatory and downstream neurons in comparison with deeper nested gamma (similar to before learning) or where gamma activity was minimal. Figure 7B shows that the downstream neuron response during the stimulus is strongest when there is shallow nested gamma and there is increased theta amplitude and strong coupling between the two frequencies. With deeper nested gamma, excitatory neuron responses appear more highly synchronized and there is reduced theta/gamma coherence and a weaker downstream neuron response. When gamma is minimised to produce a very high ratio of theta to gamma there is reduced excitatory output and downstream neuron activity and theta/gamma coherence (Figure 7B). Thus for optimal coupling between gamma and theta, and to evoke maximal responses in the downstream neuron, gamma should be shallow nested on theta, producing a slightly increased theta-gamma ratio, as seen after learning.

**Figure 8:**
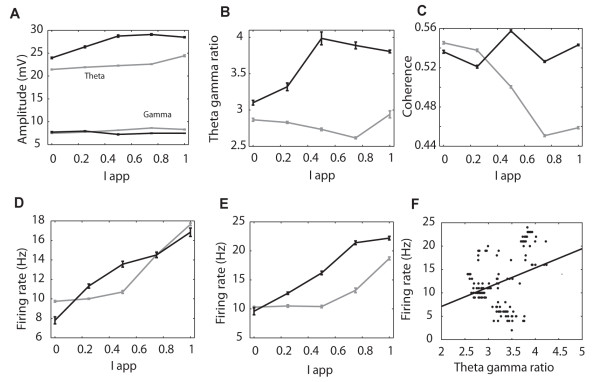
**Learning effects produced in the model by altering NMDA receptor sensitivity**. Graphs show changes (after learning = black; during learning = gray) in (A) Theta and gamma amplitude as a function of stimulus strength (I_app_). (B) Theta/gamma ratio, (C) Coherence between theta phase and gamma amplitude (D) Firing rate of the excitatory output neurons (E) Firing rate of the downstream neuron and (F) Positive correlation between downstream neuron firing rate and magnitude of the theta/gamma ratio (r = 0.34, P < 0.01). NMDA, AMPA and GABA _A _receptor coefficients after learning are the same as for shallow nested gamma in Figure 8B. For during learning NMee = 0.002 and NMes = 0.0001; after learning NMee = 0.0035 and NMes = 0.00055. Data are means ± sems from 10 averaged runs of the model. Taking an overall average across the different values of Iapp, t-tests revealed significant differences between before and after simulated learning in theta amplitude (A), t^18 ^= 81.5, p < 0.0001; gamma amplitude (B), t^18 ^= -12.1, p < 0.0001; theta/gamma ratio (C), t^18 ^= 32.02, p < 0.0001; theta/gamma coherence (D), t^18 ^= 2.6, p = 0.03; excitatory neuron firing rate (E), t^18 ^= -2.23, p = 0. 04 and the firing rate of the downstream neuron (F), t^18 ^= 13.6, p < 0.0001.

We then used the model to investigate if NMDA receptor changes alone in the network could reproduce learning-induced changes in IT theta/gamma activity. It was found that increased NMDA receptor sensitivity on and between the excitatory neurons (NMee) and between them and the slow inhibitory ones (NMes) could account for the enhanced theta amplitude without changing gamma (Figure 8A). It was possible to achieve the same outcome by combining NMDA receptor changes with increased GABA_A _receptor sensitivity between the slow inhibitory and excitatory neurons (data not shown). If the connection with the fast inhibitory neurons (NMef) was also altered this increased gamma amplitude and therefore did not replicate IT findings. Changes in the theta gamma ratio and theta/gamma coherence seen in IT recordings were also confirmed (Figures 8B and 8C). Figure 8D shows that firing rates of the excitatory neurons are unchanged following the NMDA receptor changes mimicking learning but there is nevertheless a highly significant overall increase in the firing rate of the downstream neuron (Figure 8E) and this is positively correlated with the size of the theta/gamma ratio (Pearson correlation, r = 0.34, p < 0.01 - Figure 8F).

Finally we also used the model to confirm findings in the IT that theta-gamma coupling changes were not necessarily dependent upon those of theta amplitude. To achieve this we systematically varied the strength of the connections between the slow and fast inhibitory neurons (GAsf) and within the slow inhibitory neurons (GAss). Simulations at different stimulus strengths showed that under these circumstances increased coupling strength could occur without any increase in theta amplitude (see Additional file 1, Figure S9).

### Temporal desynchronization of neuronal firing

**Figure 9:**
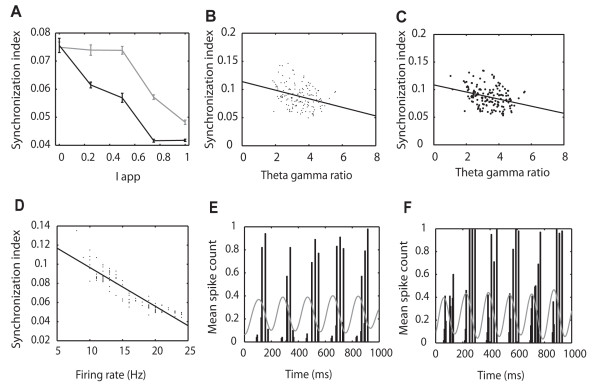
**Learning-associated desynchronization in model excitatory neurones and IT**. Graphs show (A) Significantly greater desynchronization of the 100 excitatory neurons in the model as a function of stimulus strength (Iapp) after learning (black) compared with during it (grey) (using an overall mean for all Iapp values t^18 ^= -5.30, p < 0.0001). Data are mean ± sem from 10 runs. (B) Negative correlation between synchronization and the theta/gamma ratio in MUA recordings from IT (r = -0.32, p < 0.001), (C) Negative correlation between excitatory neuron synchronization and size of the theta/gamma ratio, r = -0.42, p < 0.001, (D) Negative correlation between synchronization index and firing rate of the downstream neuron r = -0.60, p < 0.001, (E) Firing frequency distribution and theta waves generated by the model's 100 excitatory neurons in 5 ms bins for 1s after stimulus onset during learning and (F) after learning (Iapp = 0.8). After learning more time bins during theta waves have active neurons compared with before learning as a result of greater desynchronization. NMDA, AMPA and GABA _A _receptor coefficients as in Figure 8.

The potentiation of downstream neuron responses predicted by the model, even in the absence of excitatory output neuron firing rate changes, suggested a temporal re-organisation of the latter might be enhancing their impact. We therefore investigated whether temporal synchronization in excitatory neuron firing was significantly altered as a result of simulated learning changes. Repeated simulations (average of 10 runs) using the model confirmed that learning produced a significantly greater desynchronization of the excitatory neuron output across a range of stimulus strengths (overall mean ± sem synchronization index before learning = 0.068 ± 0.0005 and after learning = 0.062 ± 0.001 t-test, t^18 ^= -5.3, p < 0.0001, Figure 9A). Synchronization levels were negatively correlated with the size of the theta/gamma amplitude ratio (Pearson correlation r = -0.42, p < 0.001, Figure 9B and the firing frequency of the downstream neuron (r = -0.88, p < 0.001, Figure 9D). An analysis of the distribution of spikes from excitatory neurons in the network revealed that activity occurred primarily during the peak and subsequent fall of each theta wave and that on average significantly more time bins contained spikes after changes associated with learning (mean ± sem = 4.99 ± 0.29 before learning vs 5.92 ± 0.19 after learning, 5 ms bins during each theta wave for 1s during the stimulus, t^18 ^= -2.73, p = 0.01, Figures 9E and 9F).

Since our model predicted that learning-induced changes in theta and its relationship to gamma should increase the impact of the firing of excitatory neurons on those downstream by desynchronizing their output we hypothesized that such desynchronization should also occur in MUA recordings from IT neurons. Despite the contribution of both inhibitory interneurons and output neurons to the MUA, Figure 6C shows that after learning there was indeed a significant overall desynchronization change across the 3 animals during the period of the first theta wave after stimulus onset (during learning synchronization index = 0.0871 ± 0.007 pre-stimulus and 0.0937 ± 0.01 during stimulus, t-test, t^4 ^= -1.47, p = 0.22; after learning = 0.0861 ± 0.005 vs 0.0798 ± 0.006, t^4 ^= 5.61, p = 0.005). The proportionate change in the synchronization index between during and after learning was significant (left IT: *F_1,27 _*= 9.71, p = 0.004; right IT: *F_1,39 _*= 15.17, p = 0.001 - Figure 6D). As in the model, levels of synchronization across the two hemispheres were also negatively correlated with the theta/gamma ratio (Pearson correlation, r = -0.32, p < 0.001, Figure 9C) and after learning there were significantly more 5 ms bins with spike activity across each electrode during stimulus period theta waves in the left IT (4.30 ± 0.11 per bin during learning vs 5.96 ± 0.42 per bin after learning, *F_1,27 _*= 5.21, p = 0.031, PT, p = 0.02); using only electrodes showing stimulus-evoked increased firing rates in the recording arrays). This almost achieved significance in the right IT with the ANOVA test but did so with the permutation test (5.19 ± 0.30 per bin during learning vs 5.85 ± 0.27 per bin after learning, *F_1,39 _*= 3.59, p = 0.066, PT, p = 0.04)(see Additional file 1, Figure S10). There was also a significant negative correlation between the number of bins with spike activity per theta wave and the magnitude of the synchronization index across both hemispheres (r = - 0.30, p < 0.001). Finally, actual visual discrimination performance across the three animals was significantly negatively correlated with the change in synchronization during the stimulus (left IT, r = - 0.42, p = 0.018; right IT, r = -0.35, p = 0.018 - see Additional file 1, Figure S10- individual animals A Right IT = -0.35; B left IT = -0.46*, right IT = -0.48*; C left = -0.37, right = -0.77* - *p < 0.05).

## Discussion

Overall, our results provide the first demonstration that both theta amplitude and theta-gamma coupling in IT are strongly and independently influenced by learning and may act to amplify and improve discriminability of inputs converging onto downstream neurons through a temporal desynchronization of neuronal firing. The magnitude of observed changes also correlates strongly with behavioral discrimination performance. The novel neural network model we have developed demonstrates that competitive and reciprocal coupling between fast and slow inhibitory interneurons and excitatory output neurons is important for generating theta-nested gamma and that learning-evoked changes in IT can be simulated by increasing NMDA receptor sensitivity, as in many other learning situations [22].

The time-course required for changes in theta and gamma correlates precisely with achievement of the > 80% learning criterion and since this time course was highly variable it effectively rules out any simple explanation of our findings in terms of elapsed time or stimulus repetition. Indeed, no changes were observed when animals failed to learn even after large numbers of trials. It is unlikely that there was differential attention to the visual stimuli presented following learning since animals were extensively trained to view the fixation stimulus before each trial was initiated, and latencies and magnitudes of VEPs as well as behavioral response times were unchanged. Sheep also have very limited eye movements and the receptive fields of IT neurons responding to faces and other visual stimuli are very large [20]. While we cannot completely rule out some contribution of the P100 or N300 components of the VEP to increased theta amplitude during stimulus presentations the fact that neither of their latencies not their magnitudes were significantly influenced by learning make it unlikely that they were contributing to the large increase in theta amplitude found. Indeed, even the small number of recording electrodes showing increases in theta amplitude seen during stimulus presentation before the learning criterion had been reached did not achieve significance across the three animals.

To the best of our knowledge this is the first demonstration of extensive theta-nested gamma in IT and we confirmed both the presence of coupling and learning-evoked changes as well as correlations with behavioral performance using a coherence method as well as mean vector length [11] and modulation index [13] methods. Our model shows that it can be generated by a simple network of excitatory glutamatergic pyramidal neurons and slow and fast inhibitory GABA_A _receptor interneurons that are likely to be present in this as in other brain regions [23,24]. In the model each module of one excitatory neuron and its two inhibitory neurons is capable of generating theta-nested gamma, so our findings are not dependent upon model size. That both slow and fast inhibitory interneurons are required for the generation of theta-nested gamma confirms a previous prediction [25], although differs from another study in the hippocampus suggesting that h-currents generated in oriens-lacunosum interneurons are important [26].

Although theta amplitude changes can enhance the strength of theta-gamma coupling by making theta phase more detectable [11,12] we have shown that learning effects on theta amplitude and theta gamma coupling in IT can be dissociated in terms of which theta frequencies are involved. Thus, whereas behaviorally correlated changes in theta amplitude occurred across the 4-8 Hz range, those in coupling with gamma only occurred at 6 Hz (right IT) or 8 Hz (left IT). There were also no positive correlations between changes in theta amplitude and in theta-gamma coherence. Interestingly, in comparison with lower theta frequencies (4-6 Hz), higher ones (6-12 Hz) are resistant to cholinergic drugs [27]. This might suggest a non-cholinergic mechanism for theta-nested gamma. In any event, learning evoked changes in theta amplitude and theta-gamma coupling in the IT clearly exhibit a degree of independence and may have separate functional significance. This is supported by a recent study reporting increased theta-gamma coupling in the hippocampus following item-context association learning in rats, but without changes in theta amplitude [12].

Learning evoked increases in the theta-gamma ratio in the present study were strongly correlated with both behavioural performance and also with desynchronization in both our network model and the IT. This appears contradictory with recent human findings that an increased theta-gamma ratio is found in individuals with significant cognitive impairment [19]. However, our network model shows that when large increases in this ratio are generated (by reducing gamma power), similar to those reported in humans with cognitive impairment, then this results in reduced theta amplitude changes, weaker coupling between theta and gamma and also reduced firing in both excitatory output and downstream neurons in response to stimuli. It therefore seems probable that while small increases in the theta-gamma ratio are indicative of successful learning, very large increases are more likely to reflect learning impairment.

We have found no evidence to support a key role for resetting of theta phase in cortical information processing contrary to some previous studies [1,15,16]. The virtual absence of phase resetting supports the notion that event-related potentials are not generated by the phase resetting of oscillatory activity as has been proposed [28]. A previous study has also failed to find evidence for phase-resetting by visual stimuli [29]. An obvious role for phase resetting by an external stimulus is to synchronize oscillations across a wide network; however in our IT recordings we always found very high levels of synchronization in ongoing theta activity, so there would have been no real advantage in this respect if learning had resulted in stronger phase resetting.

The lack of any alteration in the amplitude of gamma oscillations following learning is in-line with our failure to find any change in stimulus-evoked neuronal firing rates in the IT following learning. Indeed, previous research in monkeys has also failed to find evidence for reward-associated learning changes in firing rates of individual IT neurons [30] and there is evidence in both sheep [20] and monkey [31] IT for population-based encoding. There was also no evidence for learning effects on latencies or magnitudes of the P100 and N300 components of the VEP in IT so VEP changes are unlikely to have contributed to any learning effects on phase locking [16]. There was some indication that the magnitude of the P100 was increased in response to upright face pairs compared to inverted face pairs or upright non-face objects. This latter observation is similar to the face sensitive P100 in humans [32,33] and the N170 component in monkeys [34] and humans [35]. Some studies have reported increased magnitude of various VEP components as a function of face familiarity [34,36,37], and in response to visual tetanic stimulation [38], although not in the context of learning visual discrimination between pairs of objects used in the current study.

No differential learning effects were observed between the two brain hemispheres in the MUA and LFP parameters measured in current study. However, we have also analysed our LFP data using novel causality algorithm approaches and shown that learning reduces the strength of causal connections from the left to the right IT and increases the frequency of causal connections within the right IT [39]. This increased connectivity may partly explain our observation here that only the right IT shows significant strengthening of the regularity of theta phase following learning. It is notable in this context too that in general the right IT showed significantly higher levels of theta synchronization than the left.

The presence of phase-locking between theta and MUA in the IT is in agreement with a recent study in humans [21] and phase locking has been reported in other neocortical areas in monkeys [4]. There was also a tendency for this to increase in IT following learning and a recent study has reported increased phase locking of neuronal activity with theta in human temporal lobe neurons after learning [40]. The presence of extensive phase locking between theta and neuronal spiking activity in the IT provides a potential mechanism whereby altered theta activity following learning could modulate neuronal firing to produce the desynchronization effects we have observed.

Although increased theta amplitude and theta gamma coupling might have been expected to lead to tighter control and greater synchronization in neuronal firing our model shows this is not the case. Indeed, a recent study on visual cortex neurons has also reported visual stimulus-evoked decorrelation [41]. The large changes in theta activity observed would act to modulate the firing thresholds of the output neurons cells across the network. This would inevitably increase the variability in theta activity across the network leading to a wider range of firing thresholds and neurons being less likely to fire synchronously. This is supported by our model and IT findings showing that neural spike activity is more spread out in time during theta waves in the stimulus period following learning.

The prediction from our model that increased desynchronization in IT output neurons would enhance responses by downstream target neurons might also seem counter-intuitive. However, the more synchronized are the outputs from excitatory neurons converging onto a downstream neuron, the more information can potentially be lost as more excitatory post synaptic potentials (EPSPs) are generated than are necessary to cause the downstream neuron to fire. Where EPSPs generated are more separated in time they are less likely to be rendered impotent by refractory period limitations and contribute more efficiently towards eliciting responses by the downstream neuron. Spreading the temporal pattern of inputs reaching the downstream neuron would also enhance the information content of the inputs it is required to decode

It would clearly be difficult to test the above model prediction directly *in vivo *without being able to make simultaneous recordings from multiple connected neurons in say IT and the frontal cortex. However, our combined *in vivo *and model simulation findings do provide a mechanism for how learning induced changes in theta amplitude and theta-nested gamma could modulate temporal aspects of neuronal firing in neocortical networks such that downstream networks exhibit potentiated responses even in the absence of altered firing frequencies arriving from neuronal inputs.

## Conclusions

Face discrimination learning produces significant increases in the magnitude of theta amplitude and the theta-gamma ratio and the strength of theta-gamma coupling in the inferotemporal cortex. Importantly these changes are all significantly correlated with actual behavioral performance and theta amplitude and theta-gamma coupling changes appear to occur independently. Interestingly, learning did not produce significant changes in IT neuronal firing frequencies although neuronal firing was often coupled to theta-phase. The network model which we have developed to reproduce these changes suggests that a key function of such learning-evoked changes in theta amplitude, the theta-gamma ratio and theta-gamma coupling may be to increase temporal desynchronization in neuronal firing leading to optimal timing of inputs to downstream neural networks and thereby potentiating their responses. The model's efficacy was supported by the fact that this temporal desynchronization was confirmed in our IT recordings following learning. An important functional consequence of the learning evoked changes in theta and gamma we have found in may therefore be to potentiate responses by neurons in IT projection regions to learned visual stimuli through a slight temporal desynchronization of firing by IT output neurons.

## Methods

### Animals and visual discrimination training

Three female sheep were used (Ovis aries, one Clun Forest and two Dorsets). The animals were trained initially over several months to perform operant-based face (sheep) or non-face (objects) discrimination tasks with a choice being made between two simultaneously presented pictures (side by side) only one of which was associated with a food reward. The position (left or right) of the rewarded picture was randomised in each trial. During stimulus presentations animals stood in a holding trolley and indicated their choice of picture by pressing one of two touch panels located in the front of the trolley with their nose. For correct responses the food reward was delivered automatically to a hopper between the two panels. The life-sized pictures were back projected onto a screen 0.5 m in front of the animal using a computer data projector. A white fixation spot on a black background was presented constantly in between trials to maintain attention and experimenters waited until the animals viewed this spot before triggering presentation of the image pairs (since sheep don't have extensive eye movements monitoring head position is generally sufficient to establish gaze direction). The stimulus images remained in view until the animal made an operant response (generally around 1-3 s). In each case successful learning of a face or object pair required that a performance criterion of > 80% correct choice over blocks of 20-40 presentation trials was achieved consistently (Chi-square, p = 0.05 for 20 trials and 0.01 for 40 trials). By the end of training animals were normally able to reach the > 80% correct criterion after 40-80 learning trials and to maintain this performance although in a few cases learning did not occur even after 80-140 trials. Some previously learned stimulus pairs (over periods ranging from 10 days to 9 months) were also presented during subsequent electrophysiological recording experiments although the animals were mainly presented with novel stimulus pairs and neurophysiological parameters recorded before and after the learning criterion was achieved.

For each sheep recordings were made in response to up to 10 different face or non face object pairs (Sheep A: 5 novel face and 1 novel object pair; B 7 novel face pairs, 3 previously learned face pairs and one previously learned object pair; Sheep C: 2 novel face pairs and 2 previously learned face pairs. In addition following learning effects of image inversion were recorded for one face pair in both Sheep A and Sheep B to assess whether any components of the VEP were sensitive to upright faces. Learning effects were monitored over between 80-189 trials and data was collected over blocks of 20-40 trials. For the face pairs Sheep A and B were discriminating between the faces of different socially familiar or unfamiliar individuals (face identity discrimination) whereas for Sheep C discrimination was between calm and stressed face expressions in the same animal (n = 3 pairs) or in different animals (n = 1 pair). With this latter animal the calm face was the rewarded stimulus. Where novel face or object pairs were being learned during recordings the > 80% performance criterion was normally achieved in 20-80 training trials. The face and object pairs used for each of the 3 sheep are shown in Additional file 1, Figure S1.

All animal experiments were performed in strict accordance with the UK 1986 Animals Scientific Procedures Act (including approval by the Babraham Institute Animal Welfare and Ethics Committee) and during them the animals were housed inside in individual pens and able to see and communicate with each other. Food and water were available *ad libitum*. Post-surgery all animals received both post-operative analgesia treatment to minimise discomfort and antibiotic treatment to prevent any possibility of infection.

### Electrophysiological recordings and analyses of local field potentials (LFPs) and multiunit activity (MUA) in IT

#### Surgical preparation

Following initial behavioral training sheep were surgically implanted under general anesthesia (fluothane) and full aseptic conditions with either unilateral (one animal) or bilateral planar 64-electrode (for configuration see Additional file 1, Figure S6A) arrays (epoxylite coated, etched, tungsten wires with 250 μm spacing - total array area ~2 mm × 2 mm, electrode impedance ~0.2 MΩ, tip diameter ~ 1 μm, tip exposure length ~100 um) aimed at the IT. The electrode lengths varied by ~1 mm and so this combined with the tip exposure electrodes would have been recording activity across all cortical layers. Holes (0.7 cm diameter) were trephined in the skull and the dura beneath cut and reflected. Electrode arrays were placed 18-20 mm lateral to the midline, 35 mm posterior to the tip of the frontal pole and at a depth of 20-22 mm from the brain surface using a stereotaxic micromanipulator. Electrode depths and placements were calculated with reference to X-rays, as previously described [42]. They were fixed in place with dental acrylic and stainless-steel screws attached to the skull. Two of these screws acted as reference electrodes, one for each array. Electrodes were connected to 34 pin female plugs (2 per array) also cemented in place on top of the skull.

#### LFP and MUA recording protocols

Starting 3 weeks after surgery the electrodes were connected via male plugs and ribbon cables to a 128 channel electrophysiological recording system (Cerebus 128 Data Acquisition System - Blackrock Microsystems, USA) and recordings made during performance of the different face and non-face pair operant discrimination tasks. This system allowed simultaneous recordings of both neuronal spike and local event-related (LFP) activity from each electrode. Typically, individual recording sessions lasted around 30 min and animals were presented with 2-6 blocks of 20-40 trials. There was at least a week between individual recording sessions in each animal.

The LFPs were sampled at 2 kHz and MUA spikes at 30 kHz (bandpass 0.3 Hz - 7.5KHz) and digitized for storage from ~3 seconds prior to the stimulus onset to ~3 seconds after the stimulus onset (stimulus durations were generally 1-3 s). Neural recordings from our data acquisition system consisted of two distinct large raw data files, one for the LFP and the other for the MUA. We used custom Spike 2 (Cambridge Electronic Design, Cambridge, UK) scripts to translate these into text files arranged either by trial or electrode prior to further analysis.

LFP data contaminated with noise artefacts, such as from animal chewing food, were excluded as were LFPs with unexpectedly high power. For LFPs, data were analyzed during a period of 1 second before and 1 second after stimulus onset. Trend was removed before spectral analysis. Any trials having more than 5 points outside the mean ± 5 standard deviation range were discarded before the analysis. The LFPs and MUA responses were all aligned to the onset of the visual stimuli. All analyses were carried out using custom written routines in Matlab (The Mathworks Inc, Natick, MA). Use of custom spike-sorting software revealed that 1-4 single neurons were contributing to the MUA at each electrode [43].

At the end of the experiments animals were euthanized with an intravenous injection of sodium pentobarbitone and the brains removed for subsequent histological confirmation of X-rays that array placements were within the IT cortex region. The general region where electrodes were located within the IT of these animals is also shown in [39].

#### Time dependent spectrum analysis

To extract spectral content relating to time, we used a wavelet transform to disclose the time-dependent spectrum of the LFP data. The wavelet transform convolves the LFP*x*(*t*) with a mother wavelet *ψ*(*t*) [44]:

Here we use Morlet wavelet f_0 _= 0.849 defined as:

where f_0 _is the central frequency of the wavelet. If the choice of f_0 _is appropriate the second term in the bracket, which is known as the correction term, becomes negligible, thus giving a simple Morlet wavelet:

This expression shows that Morlet wavelet is a complex sine wave within a Gaussian envelope. The Fourier transform of the Morlet wavelet is:

which has the form of a Gaussian function centred at f_0_, where f_0 _determines the wave numbers within the envelope. Here f_0 _= 0.849 (around 5.3 wave numbers) and this gives a real part where the peaks next to the central peak are half its amplitude.

The wavelet transform was applied to each individual LFP trial at each electrode (for a period of 1 s either side of stimulus onset) and a final time-dependent spectrum estimated as the trial-averaged scalograms (modulus square of the wavelet transform). When comparing pre- and during stimulus theta band activity we used the amplitude of the wavelet transform at 4-8 Hz and averaged it across this band. For the gamma band, amplitude in the 30-70 Hz frequency range was analysed. Theta and gamma power were also calculated, although in our freely behaving animals we found the amplitude measure to be less variable across trials and sessions whereas power was susceptible to abrupt changes making comparisons more difficult. To determine the significance of responsiveness at each electrode a *post-hoc *t-test (with Benjamini-Hochberg correction) was used to compare the amplitude of the wavelet transform in the 500 ms pre- with the 500 ms during-stimulus periods. Amplitude changes evoked by learning were normalized by subtracting the amplitude value of the pre-stimulus period and dividing by the maximal value for each electrode. The theta/gamma ratio was also calculated as the direct ratio between the theta amplitude and gamma amplitude (or theta power and gamma power).

#### Cross-frequency coupling between theta and gamma

We used coherence analysis to measure the dependency between the signals in the two different frequency bands. This coherence analysis detected the modulation between amplitude and phase of the two band-limited signals in each frequency band. To do this we separated the raw signal into two sets of band-pass filtered signals [11]. The first set had frequencies from 30 Hz to 70 Hz, in 2 Hz steps with a 1 Hz bandwidth. This created a real-value band-pass filtered signal set ^{*x_amplitude_*(*t*)} ^in which we could extract the amplitude signal used for the gamma band. The second set of real-value band-pass filtered signals ^{*x_phase_*(*t*)} ^was created by filtering the raw signal with centre frequencies from 2 Hz to 20 Hz, in 1 Hz steps with a 1 Hz bandwidth. This set was used to extract the phase signal for the theta band. The amplitude and phase signals were then extracted by applying a Hilbert Transform to both sets to generate complex-valued analytic band-passed signals, i.e. ^{*x_amplitude_*(*t*)} ^was taken to create a set of analytic amplitude time series ^{*A*(*t*)} ^and the phase set ^{*x_phase_*(*t*)} ^was extracted to create a set of analytic phase time series ^{*φ*(*t*)}^. Using both the amplitude and phase signals the coherence  between i-th amplitude signal A(t) and j-th phase signal ^*φ*(*t*) ^was calculated by:

where  and  are the auto-spectra for the i-th A(t) and j-th ^*φ*(*t*) ^and  the cross-spectrum between them. The confidence interval for the coherence [45] is given by:

where ^*α *^is the significant level (e.g. ^*α *= 0.01^) and K is the trial number which corresponds to the disjointed number of periodograms. The phase-locking index was then measured by the coherence in the range between 0 and 1, where values close to 1 indicate a strong cross-frequency modulation. A coherence calculation was obtained at all the pair-wise frequency combinations between the two bands and a Bonferroni correction applied to the multiple comparisons over all the frequency pairs.

We confirmed that this coherence method accurately determined the extent to which gamma amplitude changes were locked to theta phase using artificial data. We generated one theta wave and gamma wave and nested (added) them together using two sine waves of 5 Hz and 50 Hz which were linearly mixed. The gamma frequency sine wave (50 Hz) had an amplitude 1/5th of the theta frequency wave (5 Hz). Using a trial length of 500 ms, 30 trials were generated with a sampling frequency of 1 kHz. White noise was then added to the mixed sine waves with a signal to noise ratio equal to -5 dB. The coherence between the theta phase and gamma amplitude was maximal when gamma was nested directly on top of theta (Additional file 1, Figure S3) confirming that our coherence measure reliably measures the strength of coupling between theta phase and gamma amplitude. However in view of recent debates over the relative merits of different methods for measuring coupling between theta phase and gamma amplitude in electrophysiological data we additionally used the mean vector length [11] and the modulation index [13] approaches for our IT data and these produced similar results to those obtained with our coherence method.

For IT recordings, theta-gamma coupling analysis was performed for all the electrodes and at each electrode the theta/gamma values were calculated for all the pairs in the theta and gamma band. In the majority of cases 40 trials were analysed in each session. Where more than 40 trials were recorded only the first 40 were analysed and where less than 40 trials occurred the coherence was normalised to match that for 40 trials to avoid any bias due to differential numbers.

#### Theta phase reset

Since a complex Morlet wavelet was used to compute the time-dependent spectrum of the LFP, the wavelet transform also provided phase information in the time-frequency domain. We therefore took out the angle of the complex wavelet transform as the instantaneous phase of LFP at each frequency. For a given trial k at time t the phase time series ^*φ*_*k*_(*t*) ^were obtained by wavelet-transforming the LFP in trial k. If an electrode exhibited phase-locking across N trials the distribution of phase should depart from uniformity and this could be tested by a Rayleigh statistic [16,46]:

Therefore the hypothesis of uniformity could be rejected at a certain significance level if phase-locking was found for that electrode. The Z-score for the Rayleigh statistic is given as *Z *= *NR*^2^.

To determine if theta band waves exhibited phase-resetting with a locked phase over trials, we calculated the Z -scores as a function of time in the during-stimulus range across all trials. An electrode recording was considered to be exhibiting phase-locking if all the samples from the time of stimulus onset at a given frequency (4 - 8 Hz) passed the criterion of the Rayleigh test (p < 0.01) across two full oscillatory cycles. A comparison was made across all the electrodes in a recording array and a Bonferroni correction applied to compensate for type-I errors.

#### Theta phase synchronization

To assess whether there was synchronization of the LFP phases, the Rayleigh statistic was also used to calculate a Z-score across all electrodes. In each trial the Z-score for theta phase (4-8 Hz) was calculated for each time point in the 500 ms pre- and the 500 ms during-stimulus periods. If > 80% of all time points across the entire 1 s period showed significant phase-locking (p < 0.05), then LFPs were considered to be synchronized in that trial. For bilateral recordings, the left and right hemispheres were analysed separately.

#### Theta phase-tightening

We calculated the Z-scores for LFP phases in 500 ms pre-stimulus and 500 ms during stimulus periods across all the electrodes in the recording array. If the Z-score was significantly higher (t test, p < 0.05) in the during-stimulus period than in the pre-stimulus period then the phase was considered to be tightened. We used the percent change from the pre- to during-stimulus period to measure altered phase tightening.

#### Visual evoked potential (VEP)

The VEP was extracted from the LFPs by trial-averaging after aligning the data to stimulus onset. Two major peaks were identified from the VEP in the initial 500 ms of stimulus presentation: a positive peak at ~100 ms (P100) and a negative peak at ~300 ms (N300). We calculated the latency for these two peaks by finding the time corresponding to the maximum and minimum peak value respectively. The amplitudes of these two peaks were calculated as their peak values after subtracting the average baseline in the 100 ms before stimulus onset.

#### MUA phase-locking with theta and firing rates

For the analysis of MUA data a Gaussian kernel with width of 30 ms was convolved to the neuronal spike train. We used the maximum peak value in the initial 500 ms of stimulus presentation to characterise MUA response latency. To calculate phase-locking of MUA on each channel to theta, the same LFP phase data at theta band were used as in the analysis of theta phase-reset. At each given frequency from 4-8 Hz, (at 1 Hz intervals) a total length of 2 s (1 s before stimulus and 1 s after stimulus) recording data was used for both LFP and MUA. LFP phases corresponding to the MUA spike time data across all the trials were then tested for analysed for whether they passed the criterion of the Rayleigh test (p < 0.05). Where they did so, this showed the distribution of phase values was not uniform at that particular electrode and frequency and that there was significant phase-locking between MUA and the theta band wave.

For firing rate changes associated with a stimulus and overall calculation was made for each channel in each block of trials (1s before vs 1 s during a stimulus). Recording channels were also sub-divided into those showing an overall increase (excited) or decrease (inhibited) in firing rate during the stimulus.

#### MUA synchronization index

We measured synchronization in MUA data by counting the spikes within a brief time window (bin width, 2.5-10 ms). This is similar to a peri-stimulus time histogram (PSTH) over all MUA channels. In each trial we produced a PSTH over all the MUA channels and normalized it by the sum of the counts in all PSTH bins. If synchronization occurred in a certain time bin there should be a high spike count for that bin. Normalization was carried out to ensure that the influence of differential firing rates was removed. We then defined a MUA synchronization index as the sum of all the normalized spike counts which exceed half of the maximum value. We calculated the synchronization index choosing a bin width of 5 ms although we also used bin sizes of 2.5 ms and 10 ms and similar trends were observed.

The synchronization index was based on the following. Suppose that the total time T is divided into small time bins *τ*(*T*/τ = *TN*), and that R spike trains are given by *X_ik _*= 0 (there is no spike) or 1 (there is at least one spike), i = 1,2,..., R, k = 1, ..., TN. We can then define:

If we then find those  (M < TN), that are larger than max(Z_k_/2), then the synchronization index *α *can be defined as:

#### Statistics

These were performed at both the population and individual animal level. For each parameter measured 2-way ANOVAs (animals and learning as factors) were first carried out for the 3 animals where recordings were made in the right IT and the 2 animals in the left IT. To test for hemisphere differences 3-way ANOVAs (animals, learning and hemisphere as factors) were conducted. In all cases where data for a particular parameter failed the Shapiro-Wilks normality test an additional permutation analysis was carried out using 1000 replacements. Additionally, analyses within animals were carried out using t-tests corrected for multiple comparisons. Correlations between electrophysiological parameters and behavioral performance were carried out using Pearson tests.

### Network model

We constructed an excitatory-inhibitory network comprising three populations of neurons: 100 excitatory (pyramidal) neurons, 50 inhibitory fast (inter) neurons and 50 inhibitory slow (inter)neurons. Similar models using fast and slow GABA_A _kinetics have been investigated for hippocampal neurons [25]. The size of the network chosen was based on the number of neurons typically recorded by our array electrodes. Each set of neurons obeys the following integrate and fire equation:

where *^C_e_^, ^C_I _^*are the capacitances for excitatory and inhibitory neurons, and *^I_e_^*, *^I_If _^*and *^I_Is _^*represent the background currents for these three kinds of neurons, excitatory neurons(EX), fast inhibitory neurons (INf) and slow inhibitory neurons (INs). *^I_app _^*is the external input. In the model, we assume that the initial conditions of all neurons are random and the connections are all-to-all. Each cell receives AMPA and NMDA receptor mediated currents from excitatory pyramidal cells, and GABA_A _receptor mediated currents from INf neurons and INs neurons. The only exception is that INs neurons do not receive inputs from INf ones. Thus the synaptic inputs have the following general forms:

in which *^E_e_^, ^E_I _^*are reverse potentials of excitatory and inhibitory neurons, respectively; , ,  (*k,l *= *e,f,s*) are maximal channel conductances for AMPA, NMDA and GABA_A _receptors, respectively. An action potential is discharged when the membrane potential reaches a firing voltage threshold *^V_th_^*. Then the membrane potential is reset to *^V_reset _^*and stays there for an absolute refractory period *^τ_ref_^*. For EX cells, the parameters in the model are *^V_th _^*= -52 mV, *^V_reset _^*= -59 mV, *^τ_ref _^*= 2 ms, *^C_e _^*= 0.5 nF,  = 0.025*^μ^*S, *^V_eL _^*= -70 mV, the excitatory reverse potential *^E_e _^*= 0 mV. For inhibitory cells, we set *^V_th _^*= -52 mV, *^V_reset _^*= -60 mV, *^V_IL _^*= -65 mV, *^C_I _^*= 0.2 nF,  = 0.02*^μ^*S. The refractory time *^τ_ref _^*= 1 ms. *B*(*V*) represents the magnesium block depending on the relevant potential and it is calculated as *B*(*V*) = 1/[1 + exp(-0.062*V*)/3.57]. The inhibitory reverse potential *^E_I _^*= -70 mV. The gating variables  and  are described by two first-order kinetics [47]:

with *l *= *AM,NM *where *^t_j _^*is the presynaptic spike time. For channel parameters, we use ^*α_x,AM _*= 1 ^(in dimensionless), *τ_x,AM _*= 0.05 msec, *α_s,AM _*= 1.0 msec^-1 ^*τ_s,AM _*= 2.0 msec for AMPA receptors, and ^*α_x,NM _*= 1 ^(in dimensionless), *τ_x,NM _*= 2 msec *α_s,NM _*= 1.0 msec^-1 ^*τ_s,NM _*= 80 msec for NMDA receptors. The inhibitory postsynaptic current (IPSP) from slow and fast interneurons is mediated by the GABA_A _receptor. The gating variables  and  obey simple first-order kinetics [48]:

Here the superscript in  indicates that the increment of  by a spike should be calculated using the value of  immediately before the spike on the right hand side of the equation:

For the fast GABA_A _channel, we chose *τ_I,f _*= 9 ms and *α_I,f _*= 1 ms^-1^. For the slow GABA_A _channel, *τ_I,s _*= 50 ms and *α_I,s _*= 0.2 ms^-1^. In the simulation, spikes in all presynaptic neurons are connected to a convergent neuron. For the background current of EX cells, we set  where *ξ_i_*(*t*) is white noise with variance *σ_e _*= 0.01. For inhibitory cells, we set the background currents fixed and homogenous,  for INf cells, and  for INs cells.

#### Parameters and analytical methods used in application of the model

To generate post learning effects the following coefficient values were used for the different sites of AMPA (AM), NMDA (NM) and GABA_A _(GA) receptors (e = excitatory neuron, s = slow inhibitory neuron and f = fast inhibitory neuron): AMee = 0.02; AMef = 0.08; AMes = 0.0005; NMee = 0.0035; NMef = 0.001; NMes = 0.00055; GAff & GAss = 0.08; GAfe = 0.015; GAse = 0.06; GAsf = 0.03. For pre-learning only the values of two NMDA receptor coefficients were reduced: NMee to 0.002 and NMes to 0.0001. For parameters that generated different theta/gamma patterns (Figure 7), the shallow nested gamma was generated using the post learning parameters above. The parameters for deep nested gamma were the same as the shallow nested case except that GAfe = 0.045. The minimal gamma used the same parameters as the shallow nested gamma except that GAse and GAsf = 0.12. All the methods for calculating theta/gamma parameters were the same as for the data from IT recordings.

## Authors' contributions

KMK conceived and designed the IT recording experiments and carried them out with HF and AUN. Data analysis was performed by YZ, AUN, HF and KMK. JF and XZ conceived and designed the neural network model with contributions from YZ and KMK. Model simulations and analyses were performed by YZ and XZ. The paper was written by KMK, JF and YZ with technical contributions from XZ, HF and AUN. All authors read and approved the final manuscript.

## Supplementary Material

Additional file 1 **Figure S1**. Face and object stimulus pairs used in each of the three sheep.
Title: Figure S2.
Description: Theta and gamma power during a stimulus.
Title: Figure S3.
Description: Theta phase/gamma amplitude coupling of simulated data.
Title: Figure S4.
Description: Averaged visual evoked potentials (VEPs) from IT recordings.
Title: Figure S5.
Description: Examples of IT MUA phase locking to theta.
Title: Figure S6.
Description: Latency and duration of theta amplitude increase in IT and network model.
Title: Figure S7.
Description: Synchronized theta waves across IT recording arrays and in the network model
Title: Figure S8.
Description: Tightening of theta phase during a stimulus after learning in IT and network model
Title: Figure S9.
Description: Model simulations showing increased theta-gamma coherence independent of increased theta amplitude.
Title: Figure S10.
Description: IT neuronal spike activity during theta waves and correlation between altered synchronization and behavior following learning. Click here for file
