# Supplementary material for: Learning alters theta amplitude, theta-gamma coupling and neuronal synchronization in inferotemporal cortex
Source: BMC Neurosci. 2011 Jun 9;12:55. doi: 10.1186/1471-2202-12-55 (PMC3123243; doi:10.1186/1471-2202-12-55)
Supplement: Additional file 1 — Figure S1. Face and object stimulus pairs used in each of the three sheep. Title: Figure S2. Description: Theta and gamma power during a stimulus. Title: Figure S3. Description: Theta phase/gamma amplitude coupling of simulated data. Title: Figure S4. Description: Averaged visual evoked potentials (VEPs) from IT recordings. Title: Figure S5. Description: Examples of IT MUA phase locking to theta. Title: Figure S6. Description: Latency and duration of theta amplitude increase in IT and network model. Title: Figure S7. Description: Synchronized theta waves across IT recording arrays and in the network model Title: Figure S8. Description: Tightening of theta phase during a stimulus after learning in IT and network model Title: Figure S9. Description: Model simulations showing increased theta-gamma coherence independent of increased theta amplitude. Title: Figure S10. Description: IT neuronal spike activity during theta waves and correlation between altered synchronization and behavior following learning. [file 1471-2202-12-55-S1.DOC]

**Additional File 1**

**Learning alters theta amplitude, theta-gamma coupling and neuronal synchronization in inferotemporal cortex**

**Keith M Kendrick^1*^, Yang Zhan^1^, Hanno Fischer****^1^, Alister U Nicol^1^, Xuejuan Zhang^2^ and Jianfeng Feng^3^**

**1** Laboratory of Molecular Signalling, Cognitive and Systems Neuroscience Group, The Babraham Institute, Cambridge, CB22 3AT, UK.

**2** Mathematical Department, Zhejiang Normal University,Zhejiang 321004, PR China.

**3** Department of Computer Science, Warwick University, Coventry CV4 7AL UK and Centre for Computational Systems Biology, Fudan University, Shanghai, PR China.

**Figure S1.** Face and object stimulus pairs used in each of the three sheep. These were presented life-sized and in colour.


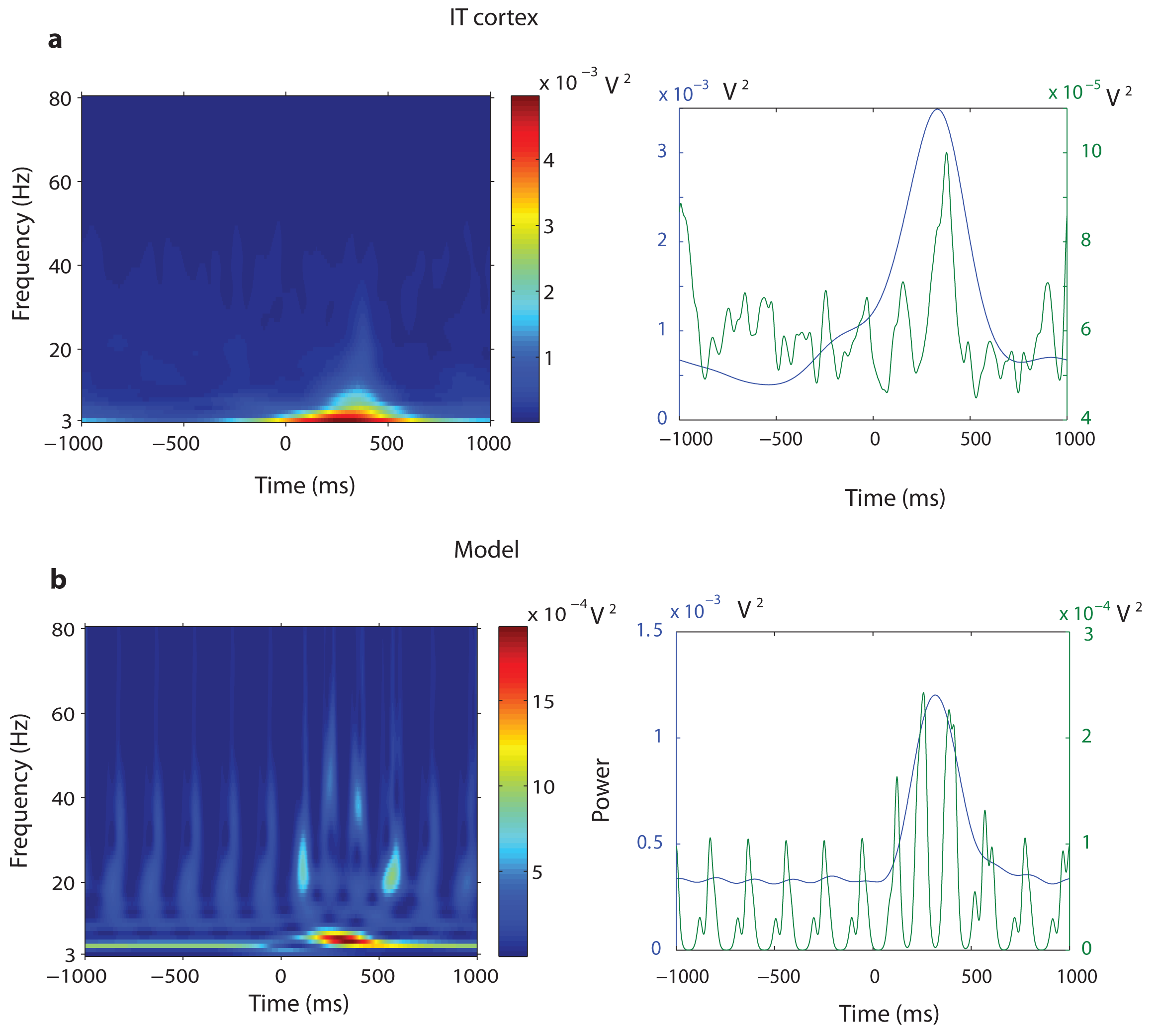


**Figure S2.** Theta and gamma power during a stimulus (a) Left, pseudocolor representation of power distribution in one channel of LFP across the theta and gamma frequency range in an IT recording before and during stimulus presentation (0-1000) and showing the greatest contribution in the theta range (4-8Hz). Right Plot of the extracted theta (blue) and gamma (red) amplitude changes during stimulus presentation with only theta being increased (data are from a typical averaged session of 40 trials – sheep B session 110305-1, channel 67). (b) The same as (**a**), but for the power spectrum produced by the LFP and extracted theta and gamma from our network model showing a similar pattern as in the IT. In this case the stimulus was a step current (Iapp = 0.8) applied from 100-400ms.


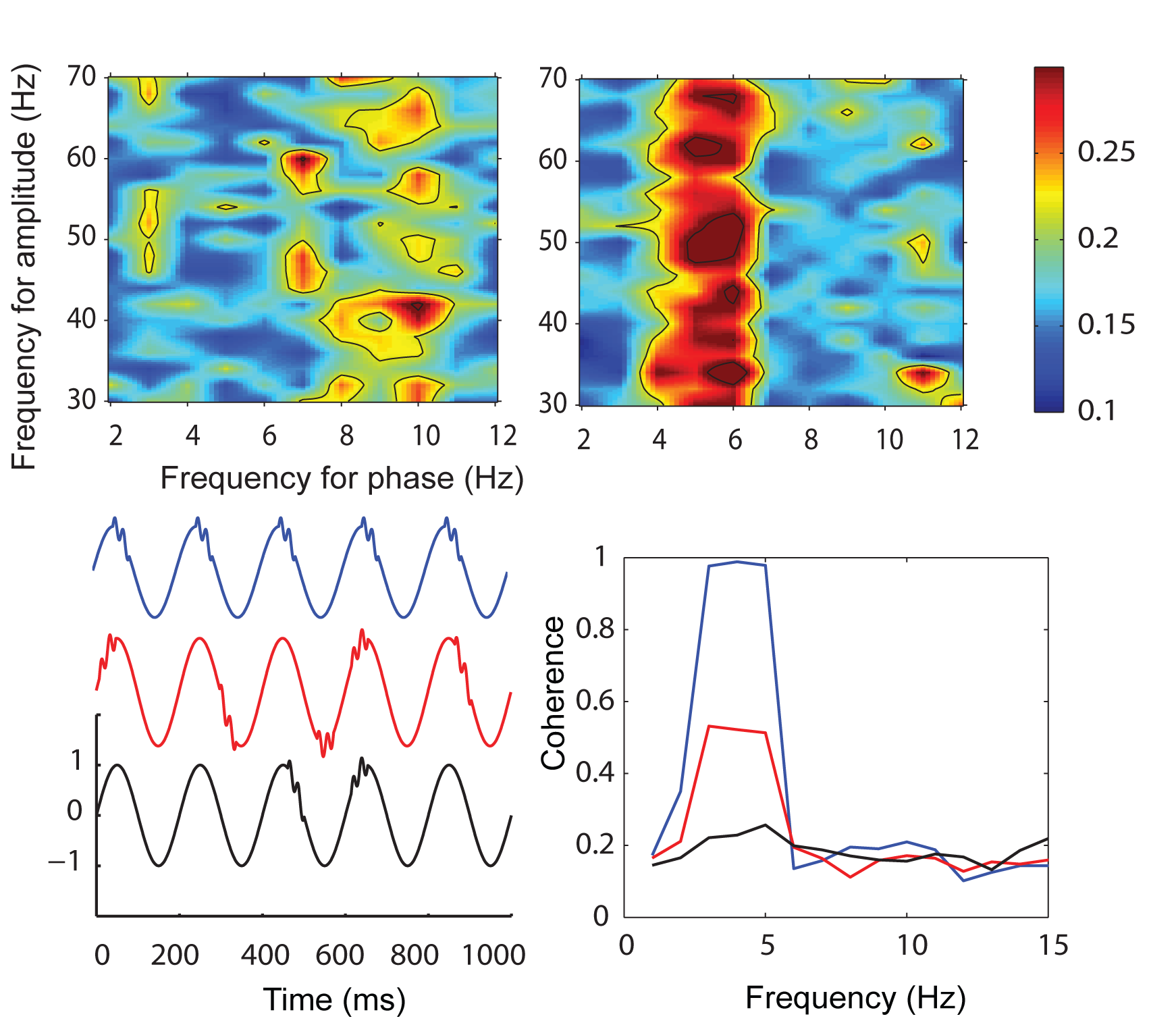


**Figure S3.** Theta phase/gamma amplitude coupling of simulated data. This shows coherence at both pre-stimulus and during stimulus periods (top two panels) and the relationship between theta phase and gamma amplitude (bottom two panels). In the top two panels 50 Hz sine waves were nested on top of 5 Hz sine waves with peak ratio 1:5. In the pre-stimulus period the amplitude of the sine wave is 1/3 of the one in the during-stimulus period. In the bottom two panels the same style of 50 Hz and 5 Hz sine waves was used with: i) Complete sets of two cycles of 50 Hz waves inserted into the same phase of each 5 Hz wave cycle (blue). ii) Complete sets of 50 Hz waves were inserted into the random phase of each 5 Hz cycle (red). iii) Incomplete sets of 50 Hz were inserted into the random phase of 5 Hz cycles in which not every 5 Hz cycle had 50 Hz waves on it (black).


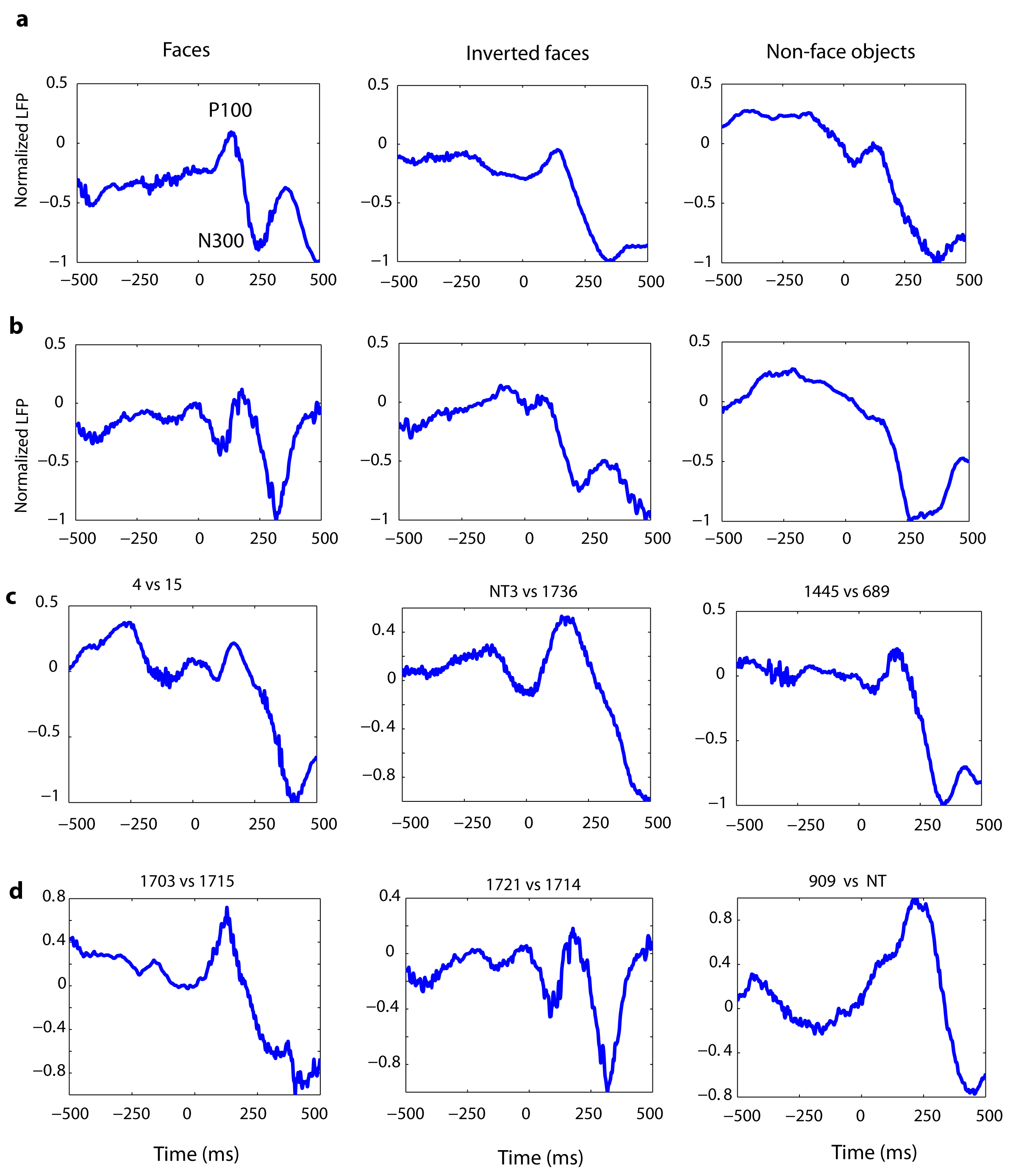


**Figure S4.** Averaged visual evoked potentials (VEPs) from IT recordings. Panels show averaged VEPS across 64 electrodes in response to upright faces, inverted faces and non-face objects in (a) The right IT in sheep A and (b) the right IT of sheep B. (c) shows VEPs for three examples of upright individual faces pairs from sheep A and (d) the same from sheep B. (a) and (b) illustrate that the P100 is consistently larger in response to upright faces than that for inverted faces or non-face objects, whereas the N300 remains relatively constant. The VEPs from upright faces in (a) and (b) are from 4-6 different face pairs, from 1 inverted face pair and from 1 non-face object pair from each animal. The LFPs are presented normalised to allow easier comparison. This was done by subtracting the baseline power during -100 to 0 ms and normalized by the absolute value of maximum negative peak.

**Figure S5.** Examples of IT MUA phase locking to theta. (a) data from sheep A session 071004 channel 52 at 4 Hz. The polar plot shows the histogram of phase distribution and the phase time series in one trial and the corresponding spike activity are shown on the right. The mean resultant length for Rayleigh test is R=0.36; (b) data from sheep B session 110305 channel 36 at 8 Hz and R=0.16. (c) data from sheep C session 160305-3 channel 101 at 6 Hz. R=0.16.


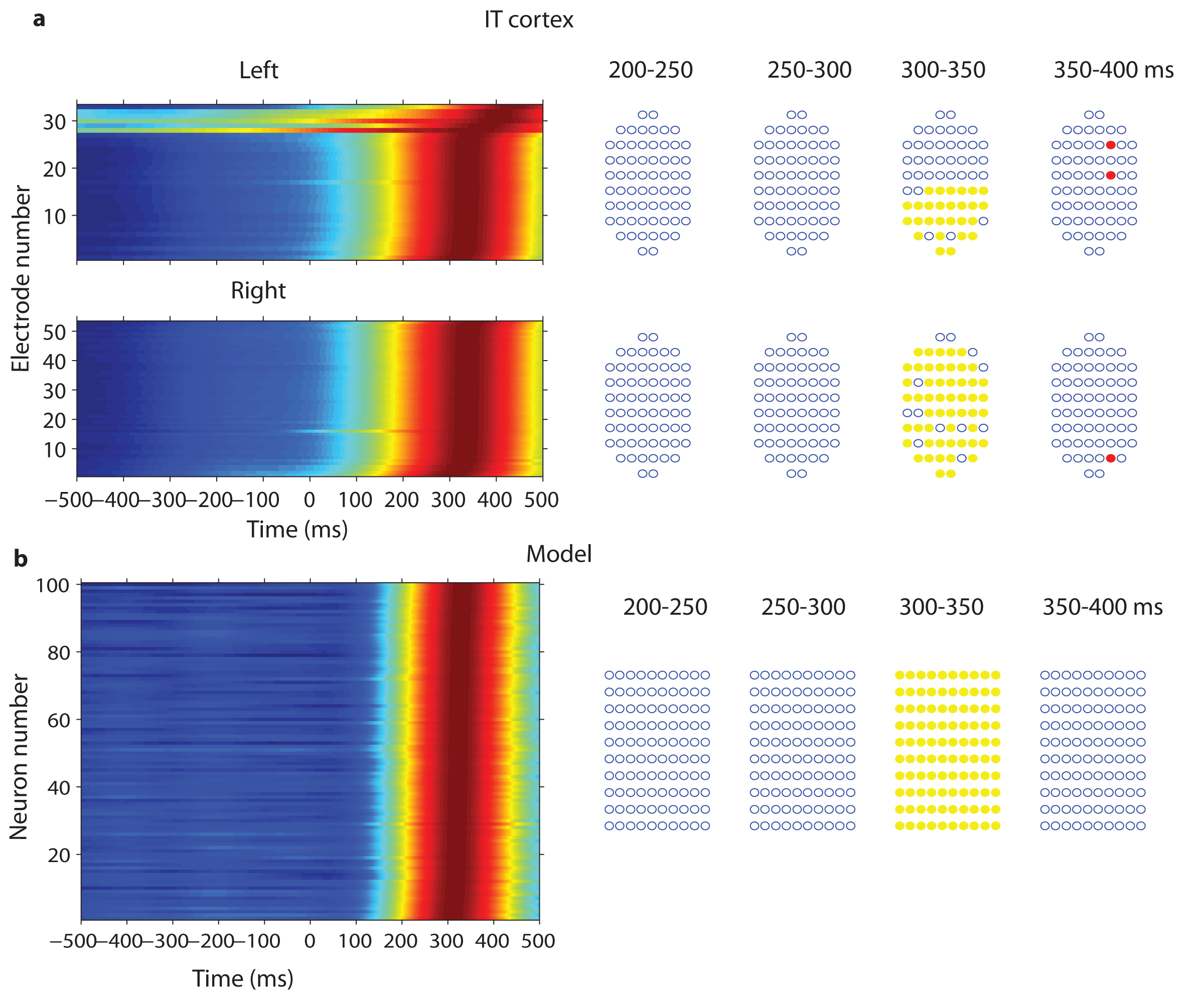


**Figure S6.** Latency and duration of theta amplitude increase in IT and network model (a) Left, pseudocolor representation of an example of the distribution of theta power for electrodes with a significant theta amplitude increase in left and right IT during stimulus presentation (from time 0) (sheep B, session 110305-1). Right, yellow and red colours in the 64-electrode grids (set out in the array configuration used in this study) indicate latency for the peak theta response for each electrode and can be seen to be primarily 300-350ms. (b) Same as (a**)**, but for the membrane potential output of the 100 excitatory neurons in our network model. Peak theta latency is also 300-350ms.


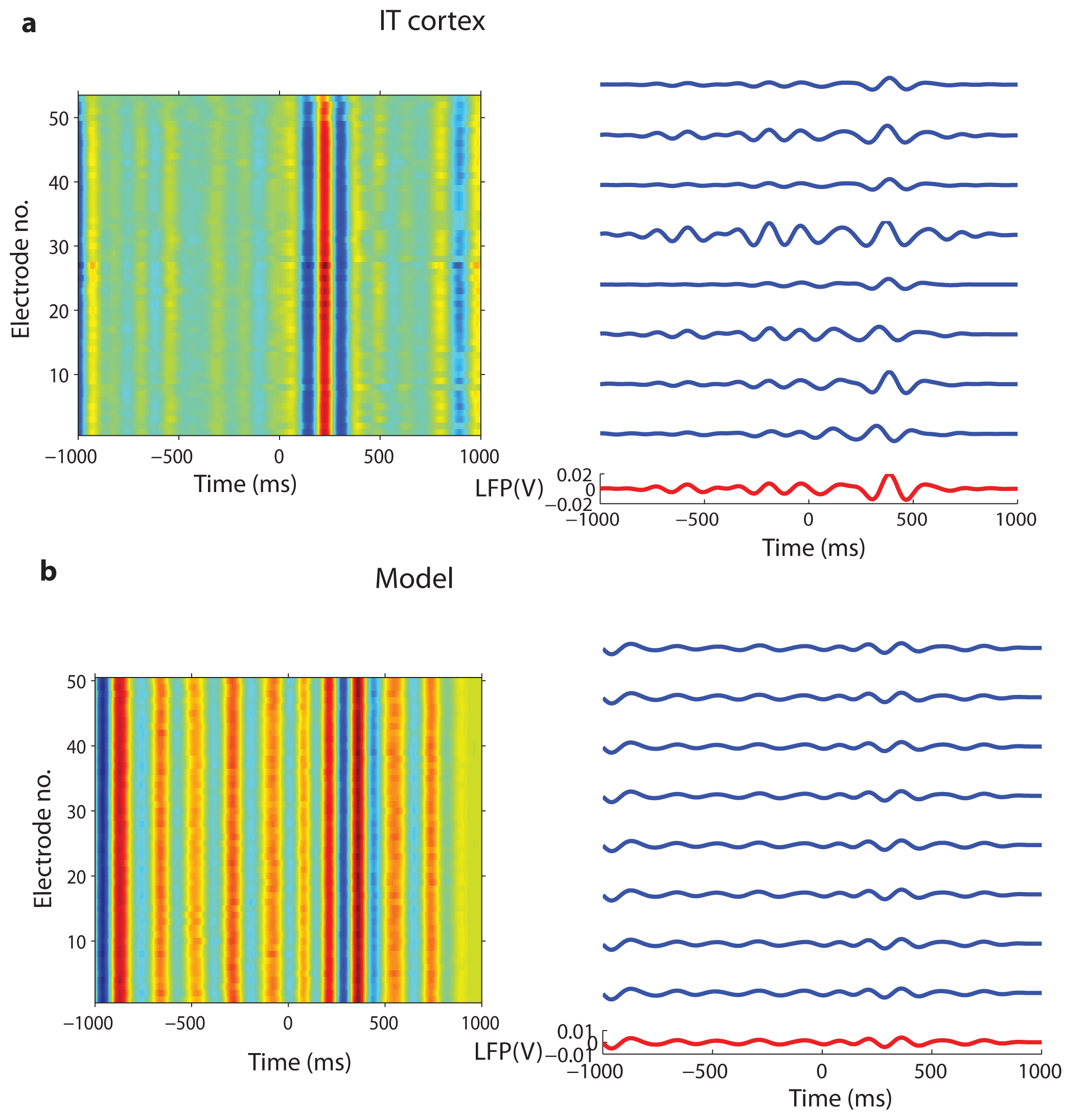


**Figure S7.** Synchronized theta waves across IT recording arrays and in the network model (a) Left, pseudocolor representation of synchronized theta wave filtered from the LFP recorded from one electrode array (sheep B, session 110305-1) during a 40 trial session. Clear bands of synchronized theta activity are seen with increased amplitude during the stimulus. Right, examples of individual electrodes (blue) averaged (red) theta band waves for all of the electrodes in the array. (b) Synchronized theta wave of 50 excitatory neurons from the network model showing the same pattern as in IT recordings.


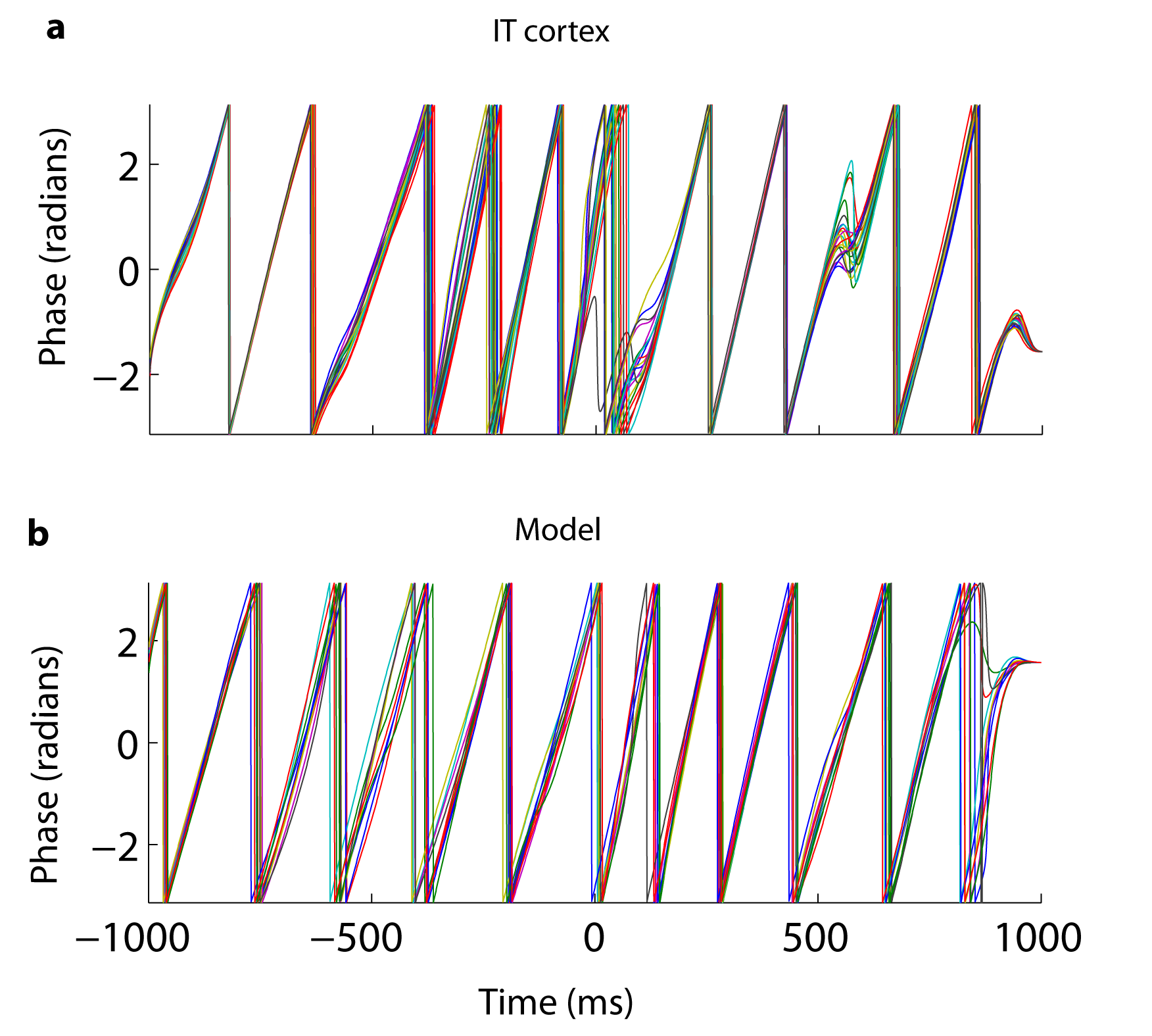


**Figure S8.** Tightening of theta phase during a stimulus after learning in IT and network model (a) Theta band phase from 10 superimposed channels (different colours) in right IT showing a tightening phase around 100-400 ms after stimulus onset(time 0) (data are from sheep B, session 110305-1). (b) Theta band phase of 10 excitatory neurons in our network model. Once again there is a phase tightening 100-400 ms after stimulus onset.


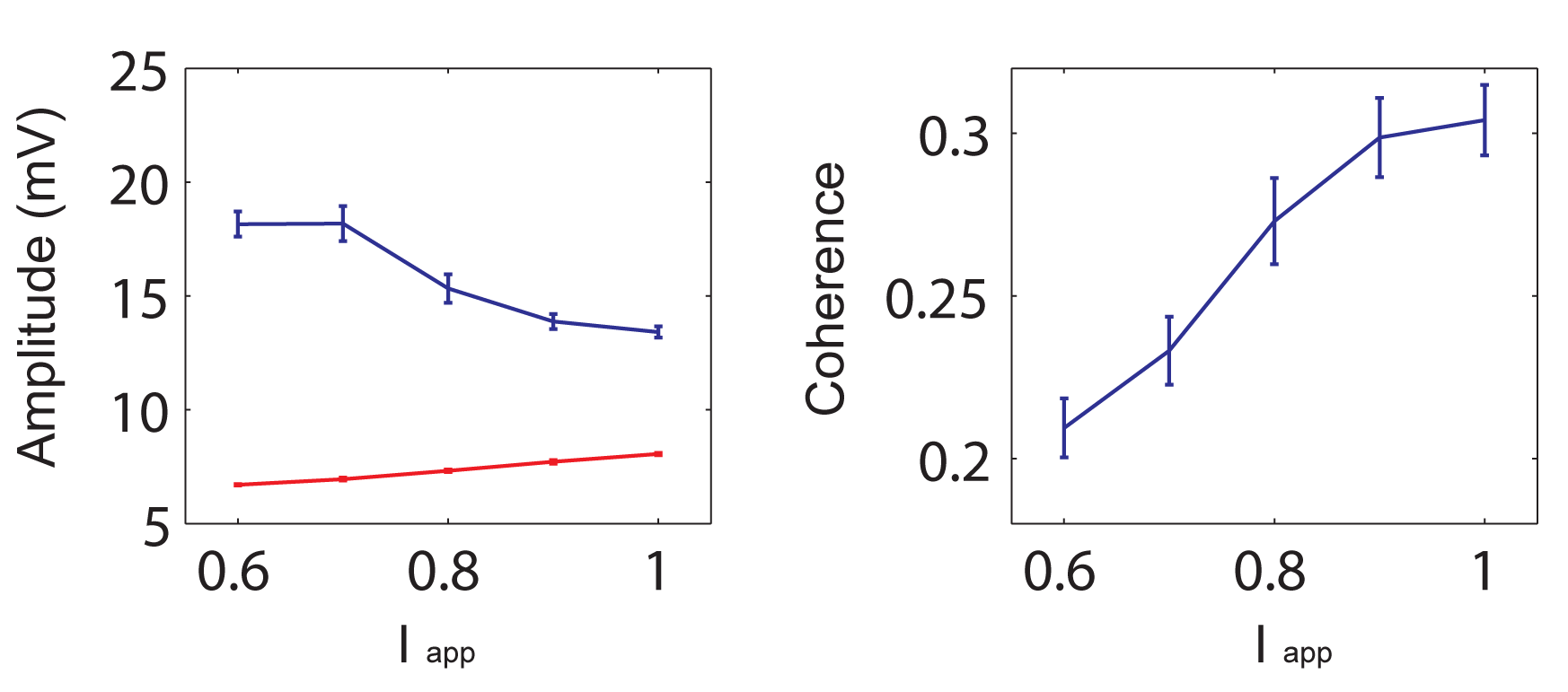


**Figure S9.** Model simulations showing increased theta-gamma coherence independent of increased theta amplitude. Graphs show mean and sem values of theta and gamma amplitude (left) and theta-gamma coherence at different applied stimulus intensities (I app) as a result of altering the strength of the connections between the slow and fast inhibitory neurons (GAsf = 0.02) and within the slow inhibitory neurons (GAss = 0.03). It can be seen that whereas theta amplitude levels actually decrease slightly with increasing stimulus strength, those of theta-gamma coherence increase. Thus coherence increases are not necessarily dependent upon those in theta amplitude. Data are from 10 runs and the other model parameters used were the same as those to simulate the before learning condition (AMee = 0.02; AMef = 0.08; AMes = 0.0005; NMee = 0.002; NMef = 0.001; NMes = 0.0001; GAff = 0.08; GAfe = 0.015; GAse = 0.06).

**
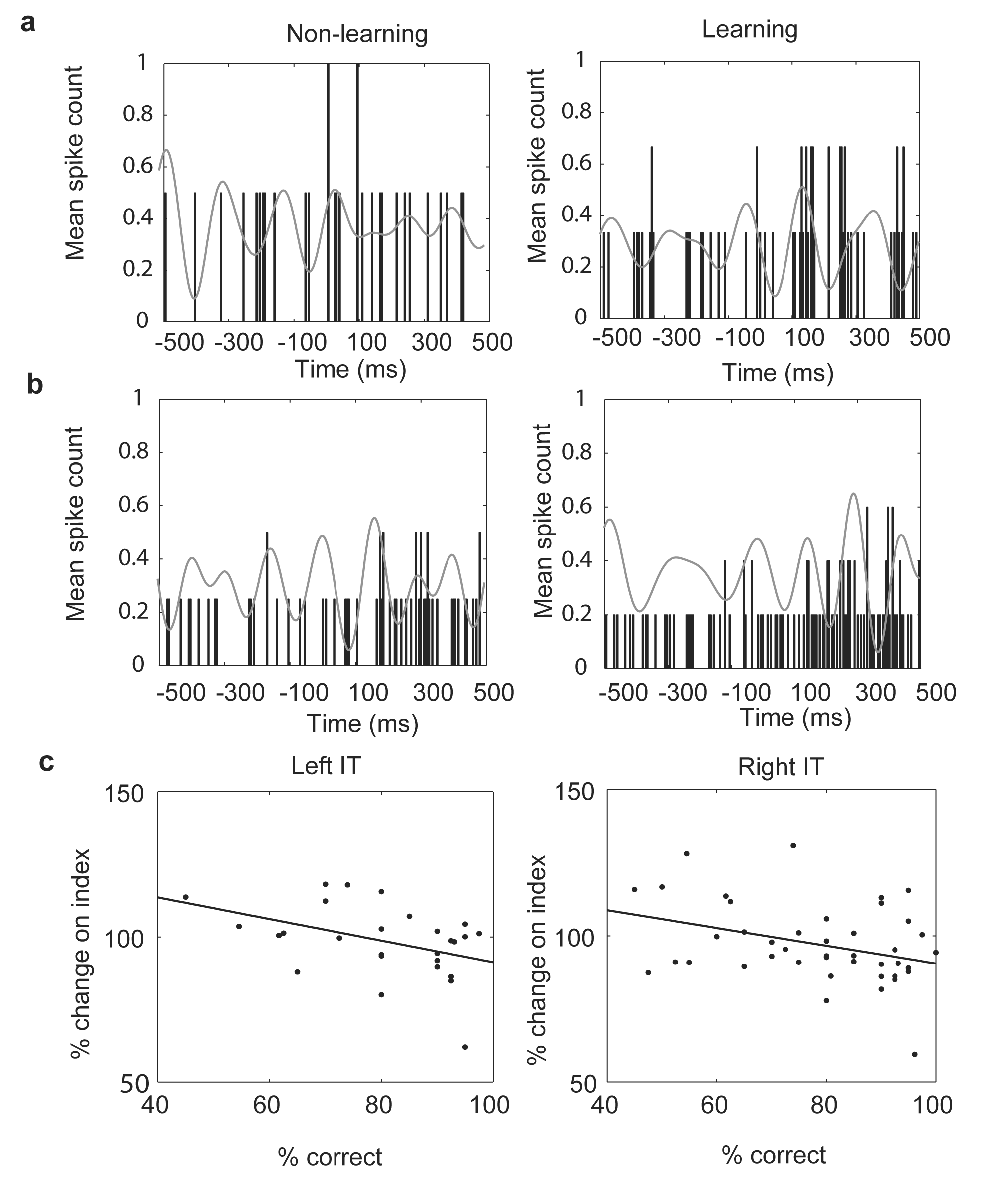
**

**Figure S10.** IT neuronal spike activity during theta waves and correlation between altered synchronization and behaviour following learning. (a) and (b) Histograms show examples of mean spike counts per electrode for 5ms time bins across 64 electrodes (where 1 represents the maximum i.e. spike present in every electrode) in the right IT for single face-pair discrimination trials (500ms before and 500ms after stimulus presentation at time 0) immediately before and after the learning criterion of >80% correct choices had been achieved. Theta wave profiles for the same trials are superimposed. (a) shows data from Sheep A for face-pair 4 vs 15, where the non-learning session (65% correct) is 100904-4 (trial 6) and the learning session (85% correct) is 100904-5 (trial 30) and (b) for face pair 1445 vs 689 the non-learning session (55% correct) is 240904-1 (trial 16) and the learning session (96% correct) is 071004-1 (trial 22). (c) Correlations between behavioural discrimination performance and changes in the synchronisation index during a stimulus (data are summarised from all three sheep – 2 with arrays in the left IT and all 3 in the right IT. For left IT: r = -0.42, p = 0.025 and for right IT: r = -0.35, p = 0.024.
